# Cancer risks of firefighters: a systematic review and meta-analysis of secular trends and region-specific differences

**DOI:** 10.1007/s00420-020-01539-0

**Published:** 2020-04-18

**Authors:** Swaantje Casjens, Thomas Brüning, Dirk Taeger

**Affiliations:** Institute for Prevention and Occupational Medicine of the German Social Accident Insurance, Institute of the Ruhr University Bochum (IPA), Bürkle-de-la-Camp-Platz 1, 44789 Bochum, Germany

**Keywords:** Firefighting, Cancer, Incidence, Mortality, Secular trend, Region-specific differences

## Abstract

**Purpose:**

The objective of this study was to conduct a systematic review and meta-analysis to evaluate the cancer risks among firefighters in the time course and from different geographical areas.

**Method:**

A PubMed search was performed to identify cohort studies about cancer risk and firefighting presented with standardized incidence ratios (SIRs) or standardized mortality ratios (SMRs). Using random-effect models, meta-relative risk estimates (mSIRs, mSMRs) and 95% confidence intervals (CI) were assessed. Cohort studies with employment starting before 1950 were classified as “old”, studies starting between 1950 and 1970 as “medium”, and later studies as “new”.

**Results:**

The general cancer risk of firefighters was similar to the general population, but mSMR decreased over time (new studies: mSMR = 0.81, 95% CI 0.70–0.92). We observed an increase of mSIR for melanoma of the skin and prostate cancer as well as a decrease of mSIR for stomach cancer with later employment onset. For those cancer sites, we did not observe a secular trend of mSMRs. Regional differences between relative cancer risks were particularly observed for bladder cancer.

**Conclusions:**

Among other things, innovative firefighting techniques and better personal protective equipment have provided a safer and healthier working environment for firefighters over time leading to a reduction of overall cancer incidence and mortality ratios. Increased general preventive medical checkups and possible additional screenings for firefighters might have led to more findings of malignant melanoma of the skin and prostate cancer in the recent past.

**Electronic supplementary material:**

The online version of this article (10.1007/s00420-020-01539-0) contains supplementary material, which is available to authorized users.

## Introduction

Firefighting is known to be a high-risk occupation. The International Agency for Research on Cancer (IARC) rated occupational exposure as a firefighter as possibly carcinogenic to humans (Group 2B) (International Agency for Research on Cancer (IARC) 2010). Firefighters are exposed to numerous carcinogens during fire suppression, but also at the fire stations. They are exposed to diesel engine exhaust if vehicles are run in closed halls or without appropriate ventilation systems (International Agency for Research on Cancer (IARC) 2010; Bott et al. 2017; Froines et al. 1987). At the fire scene, toxic and carcinogenic substances including metals, chemical substances, minerals, and various gases are released during combustion. The resulting fire smoke is a variable mixture of compounds and its toxicity varies greatly as every burning condition and burning material induces a unique pattern (Golka and Weistenhöfer 2008; Guidotti and Clough 1992).

Some meta-analyses have examined the extent of cancer risk among firefighters before and did not find higher overall cancer incidence and mortality as expected (International Agency for Research on Cancer (IARC) 2010; Jalilian et al. 2019; Sritharan et al. 2017; Crawford et al. 2017; LeMasters et al. 2006; Youakim 2006; Howe and Burch 1990), although a few single studies reported elevated overall cancer risks (Daniels et al. 2014; Glass et al. 2016; Guidotti 1993; Pukkala et al. 2014). However, meta-analyses found that firefighters were at increased risk of developing or dying from malignant melanoma of the skin (Jalilian et al. 2019; Howe and Burch 1990), multiple myeloma (LeMasters et al. 2006), mesothelioma (Jalilian et al. 2019), digestive (Jalilian et al. 2019; LeMasters et al. 2006), prostate (Jalilian et al. 2019; LeMasters et al. 2006; Sritharan et al. 2017), testicular (Jalilian et al. 2019; LeMasters et al. 2006), kidney (Youakim 2006), bladder (Jalilian et al. 2019), and thyroid cancer (Jalilian et al. 2019), as well as non-Hodgkin lymphoma (Jalilian et al. 2019; Youakim 2006; LeMasters et al. 2006).

Findings among these studies have been generally inconsistent. Reasons for this might be the lack of included non-occupational risk factors, missing specification of the exposure, period effects, and country-specific differences. All of these meta-analyses did not consider changes over time of firefighting technology, personal protective equipment (PPE), or used materials in buildings, furniture, or vehicles, which might have led to different cancer risks over the past decades. Combustion and pyrolysis products from newer building materials and furnishings (particularly polymers) are believed to be more toxic than smoke from fires in buildings built before these materials were widely used (Alarie 1985). It has been shown in a series of experimental fire tests that the highest pollutant concentrations resulted from the combustion of polymeric materials (Reisen et al. 2014). Polymers have been used in large amounts in Europe and North America since the 1950s (Guidotti and Clough 1992; Alarie 1985; Pedersen et al. 2018). However, the other studies showed that the composition of the smoke produced by fires of various kinds was similar (Austin et al. 2001b, c).

In the past, PPE of firefighters changed tremendously. The use of modern self-contained breathing apparatus (SCBA) started during the 1960s and 1970s (Misner et al. 1987), and is commonly used today by municipal firefighters, although they are not worn during the whole firefighting activity especially during overhaul (International Agency for Research on Cancer (IARC) 2010; Austin et al. 2001a). In the 1980s, modern firefighting helmets like the F1 helmet and advanced fire and heat resistant suits were introduced (Pedersen et al. 2018; Hasenmeier 2008). The cancer risk in firefighters may also vary between different geographical areas because of probably different exposure patterns depending on work activities and PPE (Howe and Burch 1990; Moher et al. 2009).

The purpose of this study was to compare cancer risks among professional firefighters from different decades and different geographic areas (North America, Europe, and Korea/Australia/New Zealand) under the assumption that firefighting cancer risks differ in the time course and between geographical areas.

## Materials and methods

### Search strategy and inclusion criteria

In March 2019, we searched the PubMed database to identify peer-reviewed original research articles available in English language about cancer risk and firefighting published until 31th December 2018 in accordance with Preferred Reporting Items for Systematic Reviews and MetaAnalyses (PRISMA) (Moher et al. 2009). The search string, following the search term from Jalilian et al. (2019), includes keywords for targeting the occupation (firefighter) and the outcome (cancer incidence or cancer mortality) is presented in Supplemental Table S1. Only articles reporting cohort studies with standardized incidence ratios (SIRs) or standardized mortality ratios (SMRs) regarding the general population as reference or case–control studies were eligible for this meta-analysis. Data extraction was performed by one author (SC) and checked by another (DT). Both authors examined the abstracts independently and agreed on the studies to be included in the meta-analysis.

**Fig. 1:**
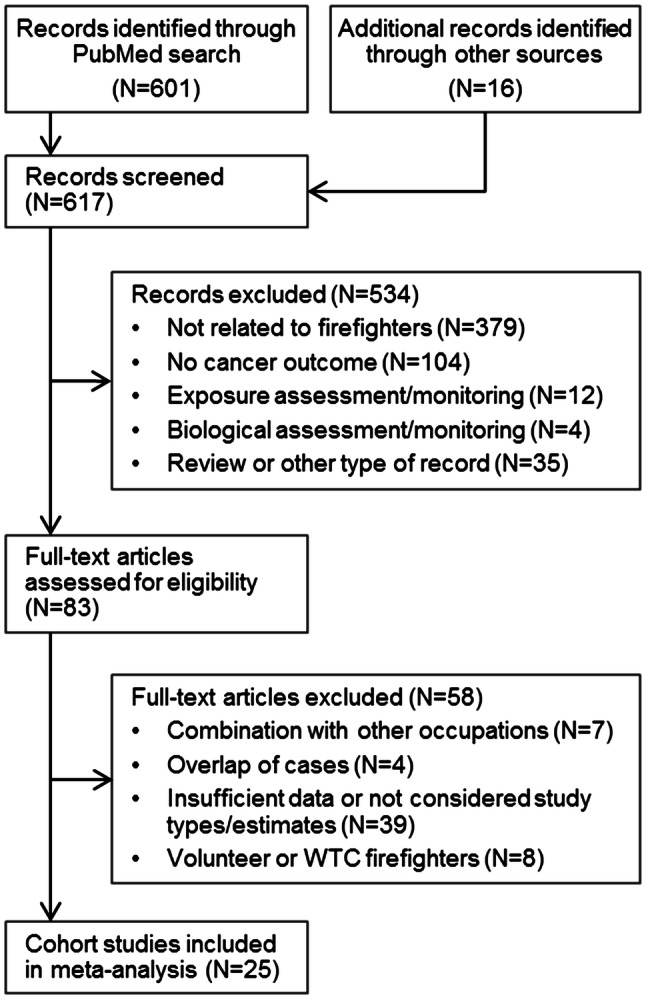
PRISMA flowchart of literature search for cancer in firefighters

The PubMed search yielded 601 articles. Additionally, the reference lists of all appropriate articles were reviewed for pertinent studies that may have been missed in the search resulting in 16 additional records. Cohort studies that examine volunteers (Glass et al. 2017), WTC-exposed firefighters (Zeig-Owens et al. 2011), or veterans (Blair et al. 1985) were excluded from the analyses because of possible different exposure patterns, less attended incidents, and the proposed healthy-volunteer effect. Case–control studies were excluded, because only a few estimates for single cancer sites existed. Hence, comparisons between regions or employment start were not feasible. When several articles provided risk measurements for the same study, only those with the broadest scope were included. For this reason, the results of four publications were not included in this meta-analysis (Firth et al. 1996; Giles et al. 1993; Heyer et al. 1990; Rosénstock et al. 1990). In total, 25 cohort studies were included in the meta-analysis (Fig. 1). We report only on cancer types which have been reported by more than one study and for male professional full-time firefighters. Cancer types were converted from earlier versions of the International Classification of Diseases (ICD) to the tenth revision (ICD-10). If appropriate, we additionally calculated estimates for all ICD-10 codes of a specific site combined, but also present the results for single ICD codes.

### Statistics

The meta-relative risk estimates (mRRs) were assessed with inverse-variance random-effects meta-analyses with Paule–Mandel heterogeneity variance estimator *τ*^2^ and presented with the according 95% confidence intervals (CI) (Paule and Mandel 1982; Veroniki et al. 2016). Greater values of *τ*^2^ depict stronger between-study variances. If *τ*^2^ = 0, the mRRs are equal to the fixed-effects estimates. For the distinction between the mRRs of different estimates, we denote mRRs based on SIRs and SMRs as mSIRs and mSMRs. The percentage of variation across studies resulting from heterogeneity rather than chance were assessed with the *I*^2^ statistic (Higgins and Thompson 2002). Heterogeneity testing based on Cochran’s *Q*. *Q* is distributed as a Chi-square statistic with *k* − 1 degrees of freedom (*k* describing the number of studies). The hypothesis of homogeneity among studies would be rejected if *Q* exceeds $\chi_{k - 1\alpha }^{2}$. The potential for publication bias was graphically explored through funnel plots and tested with the modified regression test (Lin and Chu 2018). All statistical analyses were undertaken using SAS, version 9.4 (SAS Institute Inc., Cary, NC, USA).

Subgroups reported in this analysis were defined as follows: cohort studies with employment starting before 1950 were classified as “old”, studies starting between 1950 and 1970 were classified as “medium”, and later than 31th December 1970 studies were classified as “new”. Studies located in the United States or Canada were summarized in the North America group. Studies from South Korea, Australia, and New Zealand were combined in the KOR/AUS/NZL group. The third group comprises studies from Europe, mainly from Scandinavia. Overall meta-analyses were only performed for cancer types which have been reported by more than one study. Subgroup analyses were done if data for more than one stratum existed and if cancer types have been reported by more than one study in at least one stratum. mRRs of single cancer types stratified by employment time or region were only presented in the main text if meaningful differences existed. Otherwise, results are presented in supplemental tables.

## Results

Overall, 25 articles of cohort studies were included in this meta-analysis of those 56% presenting a cancer mortality (SMR), 28% a cancer incidence outcome (SIR), and 16% both estimates (Table 1). Reported study populations were from six different countries in Europe, North America, South Korea, Australia, and New Zealand, but were mainly conducted in the United States and Canada (52% of articles). An overview of the extracted estimates from those 25 cohort studies is displayed in Supplemental Table S2.

**Table 1:** Characteristics of included cohort studies on firefighting and cancer risk

Study ID	References	Location	Outcome	Study period		Fire fighters	Sample base
				Employment	Follow-up		
1	Ahn et al. (2012)	Korea	Incidence	1980–2007	1992–2007	29,438	Registry
2	Ahn and Jeong (2015)	Korea	Mortality	1980–2007	1992–2007	29,453	Registry
3	Amadeo et al. (2015)	France	Mortality	1979–2008	1979–2008	10,829	Registry
4	Aronson et al. (1994)	Canada	Mortality	1950	1950–1989	5373	Fire department
5	Baris et al. (2001)	USA	Mortality	1925–1986	1925–1986	7789	Fire department
6	Bates et al. (2001)	New Zealand	Incidence, mortality	1977–1995	1977–1996	4221	Registry
7	Berg and Howell (1975)	USA	Mortality	1950	1950	39 deaths	Death certificate
8	Daniels et al. (2014)	USA	Incidence, mortality	1950–2009	1950–2009	29,002	Registry
9	Demers et al. (1992)	USA	Mortality	1944–1979	1945–1989	4546	Fire department
10	Demers et al. (1994)	USA	Incidence	1944–1979	1974–1989	2447	Population
11	Deschamps et al. (1995)	France	Mortality	1977–1991	1977–1991	830	Registry
12	Eliopulos et al. (1984)	Australia	Mortality	1939–1978	1939–1978	990	Registry
13	Glass et al. (2016)	Australia	Incidence, mortality	1976–2003	-2010	17,394	Registry
14	Guidotti (1993)	Canada	Mortality	1927–1987	-1987	3328	Fire department
15	Hansen (1990)	Denmark	Mortality	1970–1980	1970–1980	886	Registry
16	Kullberg et al. (2018)	Sweden	Incidence	1931–1983	1958–2012	1080	Registry
17	Ma et al. (2005)	USA	Mortality	1972–1999	1972–1999	34,796	Registry
18	Ma et al. (2006)	USA	Incidence	1981–1999	1981–1999	34,796	Registry
19	Mastromatteo (1959)	Canada	Mortality	1921–1953	1921–1953	1500	Fire department
20	Morton and Marjanovic (1984)	USA	Incidence	1963–1977	1963–1977	4 cases	Hospital
21	Musk et al. (1978)	USA	Mortality	1915–1975	1915–1975	5655	Registry
22	Petersen et al. (2018)	Sweden	Incidence	1968–2014	1968–2014	4,243	Registry
23	Pukkala et al. (2014)	Northern Europe	Incidence	1961–2005	1961–2005	16,422	Registry
24	Tornling et al. (1994)	Sweden	Mortality	1931–1983	1958–1986	1116	Registry
25	Vena and Fiedler (1987)	USA	Mortality	1950–1979	1961–2005	1867	Death certificate/registry

**Fig. 2:**
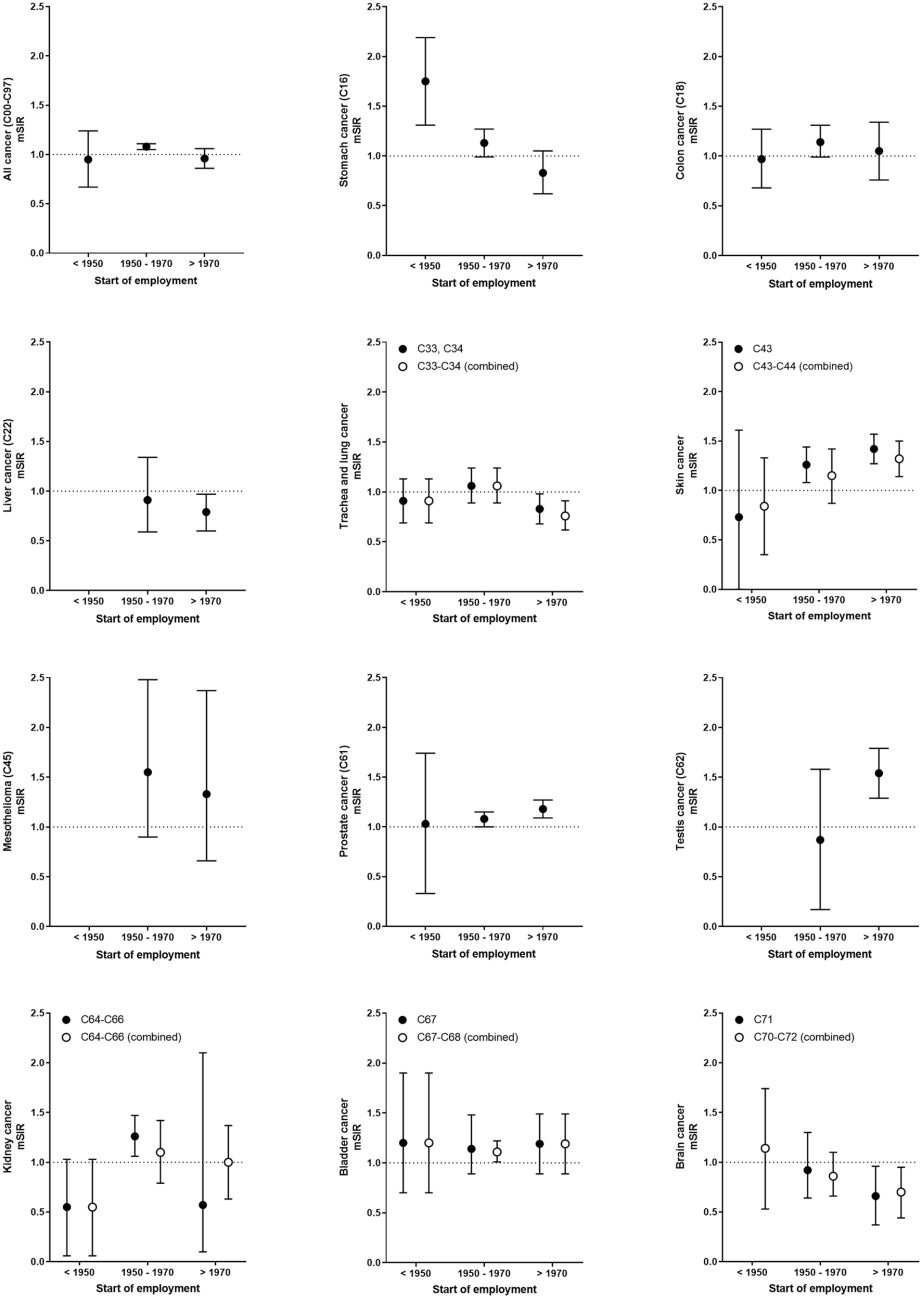
Secular trends of standardized incidence ratios of selected cancer types presented with 95% confidence intervals

Tables 2 and 3, and Supplemental Table S3 summarize the overall meta-analysis results of cancer risks of male professional firefighters from cohort studies for cancer incidence and mortality. The overall cancer mSIR of firefighters was similar to the general population and did not show a secular trend (Fig. 2). For individual cancers, we observed statistically significant elevated mSIR estimates for mesothelioma (mSIR = 1.46, 95% CI 1.01–1.90), bladder cancer (C67–C68 combined: mSIR = 1.14, 95% CI 1.04–1.23; C67: mSIR = 1.18, 95% CI 1.01–1.34), and colon cancer (mSIR = 1.11, 95% CI 1.00–1.21). We did not observe a secular trend for these cancer types. However, increased incidence risks over time exist for malignant melanoma of the skin, overall skin cancer, prostate, and testis cancer. The mSIR for stomach cancer was elevated in firefighters in the earliest employment period starting before 1950 (mSIR = 1.75, 95% CI 1.31–2.19, Supplemental Table S4) and decreases afterwards. In addition, a statistically significant reduced mSIR was observed for trachea and lung cancer in the period of employment starting after 1970. The mSIR of liver and brain cancer among firefighters was slightly lower than expected especially in the time of later employment. Other cancer sites were analyzed by only very few studies or showing no association with start of employment (Supplemental Table S4, Supplemental Fig. S1 and S2).

**Table 2:** Meta-relative risk estimates for cancer incidence

Disease (ICD-10)	# Studies	Study IDs	mSIR (95% CI)	*I*^2^ (*p* value)	*τ*^2^
All cancer (C00–C97)	9	1, 6, 8, 10, 13, 16, 18, 22, 23	1.00 (0.93–1.07)	91.3 (< 0.001)	0.010
Buccal cavity and pharynx (C00–C14)	4	10, 13, 18, 23	0.87 (0.72–1.02)	41.8 (0.161)	0.003
Lip (C00)	2	16, 23	0.84 (0.43–1.25)	0 (0.494)	0
Esophagus (C15)	8	1, 6, 8, 10, 13, 16, 18, 23	1.06 (0.76–1.36)	65.7 (0.005)	0.088
Stomach (C16)	8	1, 6, 8, 10, 13, 16, 18, 23	1.08 (0.80–1.35)	71.1 (0.001)	0.109
Small intestine (C17)	2	1, 23	1.65 (0.40–2.90)	2.2 (0.312)	0.568
Colon (C18)	6	6, 10, 13, 16, 18, 23	**1.11 (1.00–1.21)**	19.6 (0.285)	0
Colorectal combined (C18–C21)	5	1, 10, 13, 22, 23	1.08 (1.00–1.16)	0 (0.539)	0
Rectum combined (C19–C21)	8	6, 8, 10, 13, 16, 18, 22, 23	1.09 (0.99–1.19)	0 (0.819)	0
Liver and biliary passages (C22–C24)	3	1, 16, 23	0.90 (0.74–1.06)	0 (0.377)	0
Liver (C22)	4	1, 13, 18, 23	**0.81 (0.65–0.98)**	16.6 (0.309)	0
Gall bladder (C23, C24)	2	1, 23	1.16 (0.55–1.78)	40.2 (0.196)	0.100
Pancreas (C25)	8	1, 6, 10, 13, 16, 18, 22, 23	1.08 (0.88–1.28)	39.8 (0.114)	0.021
Larynx (C32)	5	1, 10, 13, 18, 23	0.88 (0.66–1.10)	0 (0.550)	0
Trachea and lung combined (C33–C34)	8	1, 6, 8, 10, 13, 16, 18, 23	0.91 (0.78–1.03)	87.7 (< 0.001)	0
Bone (C40, C41)	2	1, 18	1.38 (0.47–2.28)	0 (0.438)	0.086
Skin combined (C43–C44)	7	6, 10, 13, 16, 18, 22, 23	1.16 (0.98–1.35)	74.2 (0.001)	0.042
Malignant melanoma of skin (C43)	6	6, 10, 13, 16, 22, 23	1.19 (0.89–1.48)	78.8 (< 0.001)	0.090
Other malignant skin neoplasms (C44)	4	16, 18, 22, 23	1.10 (0.90–1.30)	63.2 (0.043)	0.026
Mesothelioma (C45)	2	13, 23	**1.46 (1.01–1.90)**	0 (0.739)	0
Soft tissue (C48, C49)	2	16, 23	1.20 (0.73–1.67)	0 (0.699)	0
Breast (C50)	4	8, 10, 13, 18	1.23 (0.27–2.19)	28.6 (0.240)	0.513
Genitourinary system (C60–C68)	2	8, 13	1.09 (0.99–1.18)	57.3 (0.126)	0.003
Male genital (C60–C63)	2	8, 13	1.10 (0.92–1.28)	85.5 (0.009)	0.016
Prostate (C61)	9	1, 6, 8, 10, 13, 16, 18, 22, 23	1.10 (0.97–1.22)	75.0 (< 0.001)	0.025
Testis (C62)	5	6, 13, 18, 22, 23	1.26 (0.87–1.65)	77.0 (0.002)	0.141
Urinary tract (C64–C68)	3	8, 10, 13	1.07 (0.92–1.22)	41.4 (0.182)	0.007
Kidney combined (C64–C66)	8	1, 6, 8, 10, 13, 16, 18, 23	0.98 (0.75–1.20)	62.6 (0.009)	0.053
Bladder combined (C67–C68)	7	1, 6, 8, 10, 13, 18, 22	**1.14 (1.04–1.23)**	0 (0.592)	0
Bladder (C67)	6	1, 6, 10, 13, 18, 22	**1.18 (1.01–1.34)**	0 (0.492)	0.005
Eye (C69)	2	8, 18	3.08 (0.00–6.62)	0 (0.461)	5.255
Brain combined (C70–C72)	7	1, 6, 10, 13, 16, 18, 23	**0.81 (0.65–0.98)**	6.2 (0.380)	0
Thyroid (C73)	5	1, 10, 13, 18, 23	1.26 (0.98–1.54)	0 (0.623)	0.011
Lymphohematopoietic (C81–C96)	4	1, 13, 16, 18	0.90 (0.63–1.17)	76.1 (0.006)	0.055
Hodgkin's disease (C81)	4	10, 13, 16, 18	0.84 (0.44–1.24)	0 (0.906)	0
Non-Hodgkin lymphoma combined (C82–C85)	6	1, 10, 13, 16, 18, 22	1.05 (0.83–1.28)	0 (0.484)	0.018
Multiple myeloma (C90)	4	10, 13, 16, 23	1.11 (0.85–1.38)	0 (0.888)	0
Leukemia (C91–C95)	9	1, 6, 8, 10, 13, 16, 18, 20, 23	1.05 (0.66–1.45)	35.4 (0.135)	0.248

Statistically significant results are marked in bold

*Study IDs* IDs of included studies in this meta-analysis as depicted in Table 1, *mSIR* meta-relative standardized incidence ratios assessed with an inverse-variance random-effects meta-analysis with Paule–Mandel heterogeneity variance estimator *τ*^2^, *CI* confidence interval, *p value p* value of heterogeneity test

**Table 3:** Meta-relative risk estimates for cancer mortality

Disease (ICD-10)	# Studies	Study IDs	mSMR (95% CI)	*I*^2^ (*p* value)	*τ*^2^
All cancer (C00–C97)	17	2, 3, 4, 5, 6, 8, 9, 11, 12, 13, 14, 15, 17, 19, 21, 24, 25	0.97 (0.89–1.05)	92.1 (< 0.001)	0.021
Buccal cavity and pharynx (C00–C14)	6	3, 5, 9, 11, 14, 17	0.97 (0.68–1.26)	68.4 (0.007)	0.044
Digestive (C15–C26)	4	11, 17, 21, 25	0.98 (0.71–1.24)	61.1 (0.052)	0.045
Esophagus (C15)	7	3, 4, 5, 8, 9, 17, 25	0.93 (0.64–1.23)	73.3 (0.001)	0.074
Stomach (C16)	11	2, 3, 4, 5, 6, 8, 9, 14, 17, 24, 25	0.94 (0.80–1.08)	48.8 (0.034)	0.009
Colon (C18)	8	3, 4, 5, 6, 9, 17, 24, 25	1.07 (0.78–1.35)	67.2 (0.003)	0.106
Colorectal combined (C18–C21)	4	2, 7, 9, 14	1.47 (0.52–2.42)	86.5 (< 0.001)	0.869
Rectum combined (C19–C21)	9	3, 4, 5, 6, 8, 9, 17, 24, 25	**1.35 (1.12–1.59)**	0 (0.692)	0.029
Liver and biliary passages (C22–C24)	3	5, 9, 25	0.95 (0.47–1.43)	0 (0.790)	0
Liver (C22)	5	2, 3, 4, 17, 24	0.84 (0.56–1.11)	75.5 (0.003)	0.035
Pancreas (C25)	8	3, 4, 5, 9, 14, 17, 24, 25	0.97 (0.73–1.22)	54.9 (0.030)	0.041
Respiratory (C30–C39)	4	11, 12, 21, 25	0.90 (0.73–1.08)	0 (0.933)	0
Larynx (C32)	3	4, 5, 9	0.59 (0.06–1.12)	0 (0.553)	0
Trachea and lung combined (C33–C34)	11	2, 3, 4, 5, 6, 8, 9, 14, 15, 17, 24	0.98 (0.86–1.11)	72.1 (< 0.001)	0.025
Skin combined (C43–C44)	7	3, 4, 5, 6, 9, 14, 17	0.87 (0.59–1.15)	0 (0.927)	0
Skin (C43, C44)	5	3, 5, 9, 14, 17	0.89 (0.59–1.19)	0 (0.823)	0
Malignant melanoma of skin (C43)	2	4, 6	0.69 (0–1.50)	0 (0.888)	0
Breast (C50)	3	3, 8, 17	3.08 (0–7.15)	10.5 (0.327)	11.926
Genitourinary system (C60–C68)	3	8, 11, 21	1.29 (0.23–2.35)	9.6 (0.331)	0.668
Prostate (C61)	9	3, 4, 5, 8, 9, 14, 17, 24, 25	1.04 (0.86–1.22)	54.8 (0.024)	0.028
Testis (C62)	2	4, 8	1.46 (0–3.18)	35.6 (0.213)	1.060
Urinary tract (C64–C68)	2	8, 9	0.72 (0–1.57)	97.0 (< 0.001)	0.345
Kidney combined (C64–C66)	8	3, 4, 5, 8, 9, 14, 24, 25	1.18 (0.42–1.94)	76.1 (< 0.001)	1.008
Bladder combined (C67–C68)	9	3, 4, 5, 6, 8, 9, 14, 17, 25	1.44 (0.82–2.06)	74.4 (< 0.001)	0.673
Bladder (C67)	7	3, 4, 5, 6, 14, 17, 25	**1.72 (1.05–2.38)**	45.6 (0.088)	0.528
Brain combined (C70–C72)	9	4, 5, 6, 9, 14, 17, 21, 24, 25	1.42 (0.90–1.93)	61.3 (0.008)	0.418
Lymphohematopoietic (C81–C96)	7	2, 3, 6, 17, 21, 24, 25	**0.76 (0.61–0.91)**	0 (0.425)	0
Hodgkin’s disease (C81)	3	4, 9, 17	0.54 (0–1.18)	54.8 (0.109)	0
Non-Hodgkin lymphoma combined (C82–C85)	4	4, 5, 9, 17	1.31 (0.92–1.70)	0 (0.446)	0
Multiple myeloma (C90)	2	4, 5	1.12 (0–2.37)	75.7 (0.042)	0.555
Leukemia combined (C91–C95, C91, C92)	6	2, 4, 5, 8, 9, 17	1.04 (0.88–1.19)	0 (0.459)	0

Statistically significant results are marked in bold

*Study IDs* IDs of included studies in this meta-analysis as depicted in Table 1, *mSMR* meta-relative standardized mortality ratios assessed with an inverse-variance random-effects meta-analysis with Paule–Mandel heterogeneity variance estimator *τ*^2^, *CI* confidence interval, *p value p* value of heterogeneity test

**Fig. 3:**
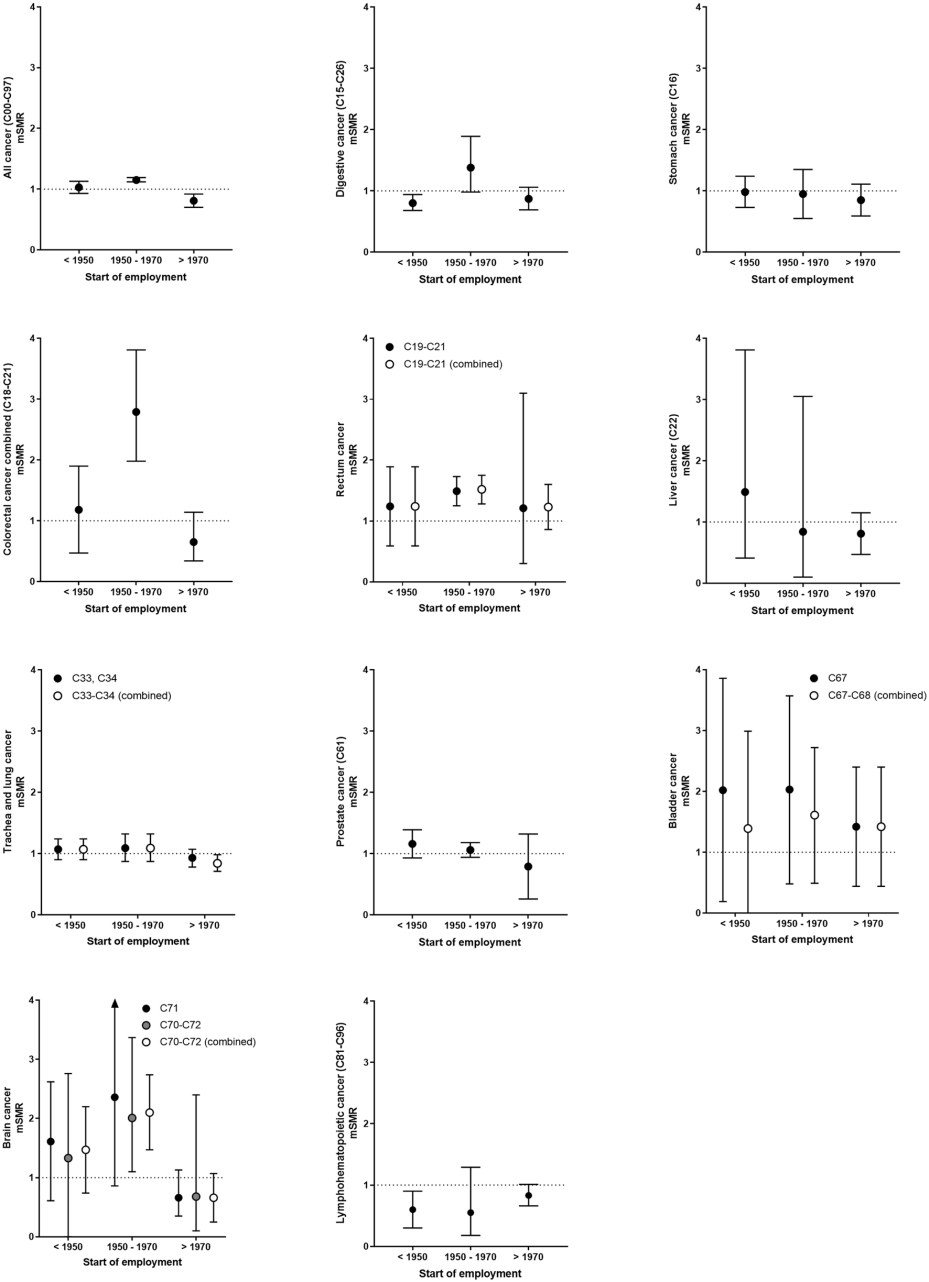
Secular trends of standardized mortality ratios of selected cancer types presented with 95% confidence intervals

The overall cancer mSMR of firefighters was similar to the general population, but decreased over time (employment start ≤ 1970: mSMR = 1.063, 95% CI 0.9–1.13 versus employment start > 1970: mSMR = 0.81, 95% CI 0.70–0.92), as shown in Fig. 3. Overall, mSMRs were increased for rectal cancer (C19–C21 combined) and bladder cancer (C67) but not when considering malignant neoplasms of bladder together with other/unspecified urinary organs (C67–C68 combined). The mSMRs for rectal and brain cancer were increased especially in studies with employment starting between 1950 and 1970. Reduced but not always statistically significant mSMRs were observed for stomach, liver, trachea and lung, prostate, and brain cancer in the period of later employment. For stomach, liver, as well as trachea and lung cancer, findings were consistent across study types revealing a decreasing mSMR with later employment. However, the mSIR of prostate cancer increased over time, whereas its mSMR decreased.

Table 4 depicts the mSIR and mSMR results for selected cancer types stratified by region. The bladder mSIRs (C67 and C67–C68) were statistically significant in USA/Canada but not in Europe or KOR/AUS/NZL. In KOR/AUS/NZL, the estimates for malignant melanoma of the skin (mSIR = 1.43, 95% CI 1.27–1.58), prostate cancer (mSIR = 1.23, 95% CI 1.11–1.34), and testis cancer (mSIR = 1.47, 95% CI 1.10–1.83) were elevated. The mSIR of pancreas cancer was statistically significant increased only in Europe (mSIR = 1.23, 95% CI 1.01–1.45). In contrast, lung cancer mSIR was reduced in KOR/AUS/NZL (mSIR = 0.83, 95% CI 0.68–0.98). Overall, mSMR were decreased in KOR/AUS/NZL studies (mSMR = 0.78, 95% CI 0.59–0.97) in comparison to studies from North America (mSMR = 1.03, 95% CI 0.94–1.12). In addition to the increased bladder cancer mSIRs in North American studies, we only observed an increased SMR for bladder cancer (C67) but not for cancer of the bladder and other/unspecified urinary organs (C67-C68) in North American studies. The mSMR of pancreas or prostate cancer did not differ between regions, but stomach as well as trachea and lung cancer mSMRs were lowest in studies from KOR/AUS/NZL. Overall, lymphohematopoietic cancer SMR was lower than expected in all studies, but more pronounced in studies from North America. There were no meaningful differences between study regions for other cancer types (Supplemental Table S5). Heterogeneity is present between the studies. Especially studies with higher standard error will tend to report SIR or SMR more extreme for overall cancer (Supplemental Fig. S3).

**Table 4:** Meta-analysis results of selected cancer types for cancer incidence and mortality stratified by region

Disease (ICD-10 code)	Region	# Studies	Study IDs	mRR (95% CI)	*I*^2^ (*p* value)	*τ*^2^
Cancer incidence						
All cancer (C00–C97)	Overall	9	1, 6, 8, 10, 13, 16, 18, 22, 23	1.00 (0.93–1.07)	91.3 (< 0.001)	0.010
	USA + Canada	3	8, 10, 18	1.01 (0.84–1.18)	96.9 (< 0.001)	0.021
	Europe	3	16, 22, 23	0.98 (0.82–1.14)	90.7 (< 0.001)	0.018
	KOR/AUS/NZL	3	1, 6, 13	1.02 (0.94–1.10)	58.7 (0.089)	0.003
Stomach (C16)	Overall	8	1, 6, 8, 10, 13, 16, 18, 23	1.08 (0.80–1.35)	71.1 (0.001)	0.109
	USA + Canada	3	8, 10, 18	0.98 (0.46–1.50)	87.9 (< 0.001)	0.156
	Europe	2	16, 23	1.45 (0.67–2.23)	74.6 (0.047)	0.281
	KOR/AUS/NZL	3	1, 6, 13	0.93 (0.77–1.10)	0 (0.894)	0
Pancreas (C25)	Overall	8	1, 6, 10, 13, 16, 18, 22, 23	1.08 (0.88–1.28)	39.8 (0.114)	0.021
	USA + Canada	2	10, 18,	0.71 (0.25–1.18)	48.9 (0.162)	0.029
	Europe	3	16, 22, 23	**1.23 (1.01–1.45)**	0 (0.530)	0.005
	KOR/AUS/NZL	3	1, 6, 13	1.05 (0.71–1.39)	0 (0.908)	0
Trachea and lung combined (C33–C34)	Overall	8	1, 6, 8, 10, 13, 16, 18, 23	0.91 (0.78–1.03)	87.7 (< 0.001)	0
	USA + Canada	3	8, 10, 18	0.93 (0.63–1.23)	95.5 (< 0.001)	0.062
	Europe	2	16, 23	0.95 (0.85–1.06)	22.9 (0.255)	0
	KOR/AUS/NZL	3	1, 6, 13	**0.83 (0.68–0.98)**	0 (0.417)	0
Skin combined (C43–C44)	Overall	7	6, 10, 13, 16, 18, 22, 23	1.16 (0.98–1.35)	74.2 (0.001)	0.042
	USA + Canada	2	10, 18	1.17 (0.97–1.38)	0 (0.947)	0
	Europe	3	16, 22, 23	1.02 (0.67–1.36)	85.6 (0.001)	0.079
	KOR/AUS/NZL	2	6, 13	**1.43 (1.27–1.58)**	0 (0.571)	0
Malignant melanoma of skin (C43)	Overall	6	6, 10, 13, 16, 22, 23	1.19 (0.89–1.48)	78.8 (< 0.001)	0.090
	USA + Canada	1	10	1.20 (0.60–2.30)	–	–
	Europe	3	16, 22, 23	1.00 (0.40–1.60)	89.6 (< 0.001)	0.237
	KOR/AUS/NZL	2	6, 13	**1.43 (1.27–1.58)**	0 (0.571)	0
Prostate (C61)	Overall	9	1, 6, 8, 10, 13, 16, 18, 22, 23	1.10 (0.97–1.22)	75.0 (< 0.001)	0.025
	USA + Canada	3	8, 10, 18	1.14 (0.93–1.35)	62.2 (0.071)	0.027
	Europe	3	16, 22, 23	0.99 (0.70–1.27)	90.1 (< 0.001)	0.057
	KOR/AUS/NZL	3	1, 6, 13	**1.23 (1.11–1.34)**	0 (0.903)	0
Testis (C62)	Overall	5	6, 13, 18, 22, 23	1.26 (0.87–1.65)	77.0 (0.002)	0.141
	USA + Canada	1	18	**1.60 (1.20–2.09)**		
	Europe	2	22, 23	0.87 (0.17–1.58)	86.4 (0.007)	0.204
	KOR/AUS/NZL	2	6, 13	**1.47 (1.10–1.83)**	0 (0.865)	0
Bladder combined (C67–C68)	Overall	7	1, 6, 8, 10, 13, 18, 22	**1.14 (1.04–1.23)**	0 (0.592)	0
	USA + Canada	3	8, 10, 18	**1.14 (1.04–1.25)**	0 (0.577)	0
	Europe	1	22	1.14 (0.89–1.48)	–	–
	KOR/AUS/NZL	3	1, 6, 13	1.15 (0.69–1.61)	36.9 (0.205)	0.075
Cancer mortality						
All cancer (C00–C97)	Overall	17	2–6, 8, 9, 11–15, 17, 19, 21, 24, 25	0.97 (0.89–1.05)	92.1 (< 0.001)	0.021
	USA + Canada	9	4, 5, 8, 9, 14, 17, 19, 21, 25	1.03 (0.94–1.12)	88.7 (< 0.001)	0.014
	Europe	4	3, 11, 15, 24	0.96 (0.90–1.02)	0 (0.741)	0
	KOR/AUS/NZL	4	2, 6, 12, 13	**0.78 (0.59–0.97)**	87.9 (< 0.001)	0.025
Stomach (C16)	Overall	11	2–6, 8, 9, 14, 17, 24, 25	0.94 (0.80–1.08)	48.8 (0.034)	0.009
	USA + Canada	7	4, 5, 8, 9, 14, 17, 25	1.00 (0.86–1.15)	30.7 (0.193)	0
	Europe	2	3, 24	1.17 (0.84–1.50)	0 (0.888)	0
	KOR/AUS/NZL	2	2, 6	**0.65 (0.39–0.91)**	0 (0.335)	0
Rectum combined (C19–C21)	Overall	9	3–6, 8, 9, 17, 24, 25	**1.35 (1.12–1.59)**	0 (0.692)	0.029
	USA + Canada	6	4, 5, 8, 9, 17, 25	1.31 (0.99–1.62)	0 (0.441)	0.061
	Europe	2	3, 24	1.58 (0.94–2.22)	0 (0.454)	0.094
	KOR/AUS/NZL	1	6	1.21 (0.30–3.10)	–	–
Pancreas (C25)	Overall	8	3–5, 9, 14, 17, 24, 25	0.97 (0.73–1.22)	54.9 (0.030)	0.041
	USA + Canada	6	4, 5, 9, 14, 17, 25	0.90 (0.60–1.21)	55.0 (0.049)	0.054
	Europe	2	3, 24	1.20 (0.89–1.52)	0 (0.350)	0
	KOR/AUS/NZL	0	–	–	–	–
Lung combined (C33–C34)	Overall	11	2–6, 8, 9, 14, 15, 17, 24	0.98 (0.86–1.11)	72.1 (< 0.001)	0.025
	USA + Canada	6	4, 5, 8, 9, 14, 17	1.05 (0.95–1.15)	43.3 (0.117)	0.007
	Europe	3	3, 15, 24	0.99 (0.61–1.36)	10.3 (0.328)	0.063
	KOR/AUS/NZL	2	2, 6	**0.64 (0.38–0.90)**	30.9 (0.229)	0
Prostate (C61)	Overall	9	3–5, 8, 9, 14, 17, 24, 25	1.04 (0.86–1.22)	54.8 (0.024)	0.028
	USA + Canada	7	4, 5, 8, 9, 14, 17, 25	1.08 (0.97–1.18)	0 (0.703)	0
	Europe	2	3, 24	0.83 (0.18–1.49)	82.6 (0.016)	0.165
	KOR/AUS/NZL	0	–	–	–	–
Bladder combined (C67–C68)	Overall	9	3–6, 8, 9, 14, 17, 25	1.44 (0.82–2.06)	74.4 (< 0.001)	0.673
	USA + Canada	7	4, 5, 8, 9, 14, 17, 25	1.50 (0.78–2.21)	79.2 (< 0.001)	0.752
	Europe	1	3	0.73 (0.41–1.21)	–	–
	KOR/AUS/NZL	1	6	2.73 (0.30–9.80)	–	–
Bladder (C67)	Overall	7	3–6, 14, 17, 25	**1.72 (1.05–2.38)**	45.6 (0.088)	0.528
	USA + Canada	5	4, 5, 14, 17, 25	**1.88 (1.16–2.59)**	0 (0.574)	0.437
	Europe	1	3	0.73 (0.41–1.21)	–	–
	KOR/AUS/NZL	1	6	2.73 (0.30–9.80)	–	–
Lymphohematopoietic (C81–C96)	Overall	7	2, 3, 6, 17, 21, 24, 25	**0.76 (0.61–0.91)**	0 (0.425)	0
	USA + Canada	3	17, 21, 25	**0.70 (0.50–0.90)**	0 (0.494)	0
	Europe	2	3, 24	0.80 (0.45–1.15)	66.9 (0.082)	0.018
	KOR/AUS/NZL	2	2, 6	0.86 (0.44–1.28)	0 (0.632)	0

Statistically significant results are marked in bold

*Study IDs* IDs of included studies in this meta-analysis as depicted in Table 1, *mRR* meta-relative risk estimates (cancer incidence: standardized incidence ratios; cancer mortality: standardized mortality ratios) assessed with an inverse-variance random-effects meta-analysis with Paule–Mandel heterogeneity variance estimator *τ*^2^, *CI* confidence interval, *p value p* value of heterogeneity test

## Discussion

This is the first meta-analysis exploring region-specific differences and secular trends. High heterogeneity was present in the previous meta-analyses that may be explained by these factors. We included the most recently published cohort studies on cancer risks of male professional firefighters by a systematic literature search to compare cancer risks among firefighters from different decades and different geographic areas.

Period of employment has been investigated as confounder according to cancer risks among firefighters in single-cohort studies before (Guidotti 1993; Baris et al. 2001; Kullberg et al. 2018; Petersen et al. 2018; Vena and Fiedler 1987), but was not yet subject of a meta-analysis. Here, we observed a decline of the overall cancer mSMR in the employment period greater than 1970. In accordance with a cohort study of Danish firefighters we also found an increased pancreas cancer mSIR when employment started before 1970 (mSIR = 1.22, 95% CI 1.03–1.41 vs. single study SIR = 1.63, 95% CI 1.08–2.48) in comparison to later periods (Petersen et al. 2018). An increased mSMR for bladder cancer (C67), especially in earlier employment periods, is in line with some other cohort studies (Baris et al. 2001; Guidotti 1993; Vena and Fiedler 1987). On the other hand, the changing estimates over time for malignant melanoma of the skin, prostate, testis, stomach, and lung cancer in this meta-analysis were not observed before (Baris et al. 2001; Kullberg et al. 2018; Petersen et al. 2018). We saw no elevated overall cancer mSIR in firefighters, so this meta-analysis could not confirm the reported trend of lower overall cancer incidence among firefighters employed in later periods (Kullberg et al. 2018). This might be due to the specific characteristic of that cohort study by Kullberg et al.’s finding generally very low SIRs lying outside the funnel plot of this meta-analysis (Supplemental Fig. S3).

Our results for prostate cancer with estimates for mSIR and mSMR, showing in opposed directions over time, are plausible. Prostate cancer is the second most common malignancy in men worldwide and the sixth most leading cause of cancer death (Baade et al. 2009). With the advent of Prostate-specific antigen (PSA) testing in the mid-late 1980 in the United States and other Western countries, more prostate cancers are diagnosed (Baade et al. 2009). Special screening programs for firefighters and a higher awareness of potential risks as shown in the World Trade Center Health Registry cohort (Yung et al. 2018) may result in additional PSA testing and, hence, more diagnosed prostate cancers in comparison to the general population. On the other hand, mortality rates decrease especially in developed countries because of earlier diagnosis due to PSA testing and improved treatment (Baade et al. 2009). In this meta-analysis, geographical differences in prostate cancer mSIRs might be an incidental finding, because the KOR/AUS/NZL studies presenting SIRs for prostate cancer examined only firefighters employed after the mid-1970s.

Occupational exposure as a firefighter has been classified as possibly carcinogenic to humans by IARC with strongest evidence not only on the basis of prostate cancer but also because of testicular cancer (International Agency for Research on Cancer (IARC) 2010). In accordance with earlier meta-analyses (Jalilian et al. 2019; LeMasters et al. 2006), we found an elevated mSIR for testicular cancer being more pronounced in studies with a later period of employment. The incidence rate of testicular cancer has increased especially in Western countries since the middle of the twentieth century (Manecksha and Fitzpatrick 2009). Because testicular cancer occurs among younger men with high survival, mortality studies are less relevant (Manecksha and Fitzpatrick 2009).

In the recent past, general preventive medical checkups lead to higher incidence rates of malignant melanoma of the skin as this is true for prostate cancer (Brunssen et al. 2017). Again, special screening programs for firefighters and higher participation rates may result in more diagnosed melanomas in comparison to the general population as we can see in this meta-analysis and as it has been shown before (Jalilian et al. 2019; LeMasters et al. 2006). However, the increased mSIR has not been accompanied by a corresponding increase in mSMR which is in line with data from the United States (National Cancer Institute 2018). We also found higher incidence rates of malignant melanoma of the skin in studies from Australia and New Zealand (mSIR = 1.43, 95% CI 1.27–1.58) in comparison to studies conducted in other countries (mSIR = 1.05, 95% CI 0.62–1.49) which might be rather caused by exposure to strong sunlight than occupational exposure as a firefighter (Leiter and Garbe 2008).

Although lung cancer is the leading cause of cancer related deaths worldwide (Youlden et al. 2008), earlier meta-analyses did not find any association between firefighting and lung cancer (Jalilian et al. 2019; LeMasters et al. 2006). Just a recent cohort of US firefighters from San Francisco, Chicago, and Philadelphia reported increased lung cancer morbidity and mortality risks (Daniels et al. 2014). In general, the study by Daniels et al. reported higher SIRs and SMRs than the other studies and lying outside the funnel plots (Supplemental Fig. S3). However, the firefighters in the above-mentioned study were older at end of follow-up (mean age 60 years) than in the other studies which might have also contributed to these findings. Furthermore, the rather younger age of firefighters in the later studies might have caused the statistically significant deficit in trachea and lung cancer incidence (mSIR = 0.76, 95% CI 0.62–0.91).

In accordance with the latest meta-analysis, we did not find an overall association of stomach cancer and firefighting (Jalilian et al. 2019). However, stomach cancer was more common than expected in studies with a start of employment before 1950 which is in line with a more detailed analysis in Swedish firefighters (Tornling et al. 1994). Stomach cancer has been linked to several occupational exposures, such as working in the rubber manufacturing industry, mining industry, and agricultural industry, as well as exposure to crystalline silica, hexavalent chromium, asbestos, lead compounds, and nitrate (Raj et al. 2003; Blair and Freeman 2009; Lee et al. 2016; Welling et al. 2015; Cogliano et al. 2011). Firefighters could be exposed to those compounds if the fire site holds these materials. Furthermore, “dusty occupations” could be related to stomach cancer (Raj et al. 2003) which might also apply to firefighters who get in contact with dust for example during overhaul. However, the numbers were small with 35 observed stomach cancer cases in the strata of the early employment start.

We revealed lower overall and stomach cancer mSMRs in KOR/AUS/NZL than the United States and Canada. However, studies from KOR/AUS/NZL were conducted in later periods of employment with 80% representing a start of employment after 1970 in comparison to 23% of North American studies. Hence, the difference in mSMRs between regions may result from an actual decline of the overall cancer mortality with later period of employment.

Overall, mSIR and mSMR of bladder cancer (C67) were higher than expected. We did not observe any considerable secular trend but again lower estimates in studies from other countries, in comparison to studies from North America. However, just two non-American studies reported bladder cancer mortality risks. Hence, the risk was driven by the USA/Canada studies. In contrast to the incidence studies, the mortality studies are heterogeneous, but, here, we found the highest risk for all cancer entities. Firefighters are exposed to carcinogens associated with combustion, including polycyclic aromatic hydrocarbons, which represent an important risk factor of bladder cancer (International Agency for Research on Cancer (IARC) 2010). Possible different exposure patterns depending on work activities and PPE may have caused such findings.

The limitations of the epidemiological data have to be acknowledged as well. Some of the studies examined relatively small populations of firefighters and thus have low statistical power to analyze especially rare cancer types. Additionally, the low number of eligible studies and different published diagnosis codes for cancer types (e.g., kidney cancer coded as C64, C64–C65, or C64–C66) and individually published combination of ICD codes [e.g., brain cancer (C70–C72) together with malignant neoplasm of peripheral nerves of head, face, and neck (C47) (Daniels et al. 2014)] complicate the analysis and contribute to low number of studies for each cancer type, especially after stratification by employment period and region. Therefore, it cannot be ruled out that risks will change after new studies will be published. Some studies analyzed incidence and mortality over several decades, which lead to uncertainties in the evaluation of trends in diagnosis, differences in exposure, and changes in PPE over time. Furthermore, the length of follow-up might contribute to biased findings. Especially in cohorts of younger firefighters, too short follow-up times might prevent to observe cancers associated with older age. In addition, the reader should have in mind that comparing SIRs or SMRs between groups is difficult if their confounder distributions differ (Checkoway et al. 2004). In general, publications and data from other regions are missing, e.g., Southern Europe, Asia, and South and Central America. Finally, smoking habits and other risk factors were not available in these studies.

In contrast to population-based case–control studies, it is unlikely that cohort studies were missed during the literature search which has been recently published in a letter to the editor (Casjens et al. 2019). The firefighter’s population of cohort studies is well defined, and this will lead to a better estimation of potential risks and lesser bias. Additionally, instead of the commonly used DerSimonian–Laird estimator, we used in this meta-analysis the estimator proposed by Paule and Mandel being a better alternative to estimate the between-study variance (Veroniki et al. 2016).

In this meta-analysis of 25 cohort studies of firefighters, the overall summary relative risk estimates were rather moderate with the exception of bladder cancer mortality. However, our results suggested differences of cancer mSIRs and mSMRs over time and between regions.

## Conclusion

There are secular trends and region-specific differences in the relative risks of some cancer sites of male professional firefighters. The risk estimates are rather moderate and mostly declining over time. The introduction of innovative firefighting techniques, safer PPE, better communications, and information systems, as well as changes in the awareness of hazards have provided a safer and healthier working environment for firefighters over time leading to a reduction of overall cancer SIR and SMR. The increase of general preventive medical checkups and possible additional screenings for firefighters might have led to higher rate of diagnosed prostate cancer and malignant melanoma of the skin in the recent past. However, further efforts must be made to make the job as a firefighter even safer.

## Electronic supplementary material

Below is the link to the electronic supplementary material.

Supplementary file1 (DOCX 311 kb)

## References

[CR1] Ahn Y-S, Jeong KS (2015). Mortality due to malignant and non-malignant diseases in Korean professional emergency responders. PLoS One.

[CR2] Ahn Y-S, Jeong K-S, Kim K-S (2012). Cancer morbidity of professional emergency responders in Korea. Am J Ind Med.

[CR3] Alarie Y (1985). The toxicity of smoke from polymeric materials during thermal decomposition. Annu Rev Pharmacol Toxicol.

[CR4] Amadeo B, Marchand J-L, Moisan F, Donnadieu S, Gaëlle C, Simone M-P, Lembeye C, Imbernon E, Brochard P (2015). French firefighter mortality: analysis over a 30-year period. Am J Ind Med.

[CR5] Aronson KJ, Tomlinson GA, Smith L (1994). Mortality among fire fighters in metropolitan Toronto. Am J Ind Med.

[CR6] Austin CC, Dussault G, Ecobichon DJ (2001). Municipal firefighter exposure groups, time spent at fires and use of self-contained-breathing-apparatus. Am J Ind Med.

[CR7] Austin CC, Wang D, Ecobichon DJ, Dussault G (2001). Characterization of volatile organic compounds in smoke at experimental fires. J Toxicol Environ Health Part A.

[CR8] Austin CC, Wang D, Ecobichon DJ, Dussault G (2001). Characterization of volatile organic compounds in smoke at municipal structural fires. J Toxicol Environ Health Part A.

[CR9] Baade PD, Youlden DR, Krnjacki LJ (2009). International epidemiology of prostate cancer: geographical distribution and secular trends. Mol Nutr Food Res.

[CR10] Baris D, Garrity TJ, Telles JL, Heineman EF, Olshan A, Zahm SH (2001). Cohort mortality study of Philadelphia firefighters. Am J Ind Med.

[CR11] Bates MN, Fawcett J, Garrett N, Arnold R, Pearce N, Woodward A (2001). Is testicular cancer an occupational disease of fire fighters?. Am J Ind Med.

[CR12] Berg JW, Howell MA (1975). Occupation and bowel cancer. J Toxicol Environ Health.

[CR13] Blair A, Freeman LB (2009). Epidemiologic studies in agricultural populations: observations and future directions. J Agromed.

[CR14] Blair A, Walrath J, Rogot E (1985). Mortality patterns among US veterans by occupation. I. Cancer. J Natl Cancer Inst.

[CR15] Bott RC, Kirk KM, Logan MB, Reid DA (2017). Diesel particulate matter and polycyclic aromatic hydrocarbons in fire stations. Environ Sci Process Impacts.

[CR16] Brunssen A, Waldmann A, Eisemann N, Katalinic A (2017). Impact of skin cancer screening and secondary prevention campaigns on skin cancer incidence and mortality: a systematic review. J Am Acad Dermatol.

[CR17] Casjens S, Brüning T, Taeger D (2019). Meta-analysis of cancer risks of professional firefighters. Int J Cancer.

[CR18] Checkoway H, Pearce N, Kriebel D (2004). Research methods in occupational epidemiology.

[CR19] Cogliano VJ, Baan R, Straif K, Grosse Y, Lauby-Secretan B, El Ghissassi F, Bouvard V, Benbrahim-Tallaa L, Guha N, Freeman C, Galichet L, Wild CP (2011). Preventable exposures associated with human cancers. J Natl Cancer Inst.

[CR20] Crawford JO, Winski T, McElvenny D, Graveling R, Dixon K (2017) Firefighters and cancer: the epidemiological evidence. Research Report TM/17/01 (Research Avenue North, Riccarton, Edinburgh, EH14 4AP, Institute of Occupational Medicine 2017)

[CR21] Daniels RD, Kubale TL, Yiin JH, Dahm MM, Hales TR, Baris D, Zahm SH, Beaumont JJ, Waters KM, Pinkerton LE (2014). Mortality and cancer incidence in a pooled cohort of US firefighters from San Francisco, Chicago and Philadelphia (1950–2009). Occup Environ Med.

[CR22] Demers PA, Checkoway H, Vaughan TL, Weiss NS, Heyer NJ, Rosenstock L (1994). Cancer incidence among firefighters in Seattle and Tacoma, Washington (United States). Cancer Causes Control.

[CR23] Demers PA, Heyer NJ, Rosenstock L (1992). Mortality among firefighters from three northwestern United States cities. Br J Ind Med.

[CR24] Deschamps S, Momas I, Festy B (1995). Mortality amongst Paris fire-fighters. Eur J Epidemiol.

[CR25] Eliopulos E, Armstrong BK, Spickett JT, Heyworth F (1984). Mortality of fire fighters in Western Australia. Br J Ind Med.

[CR26] Firth HM, Cooke KR, Herbison GP (1996). Male cancer incidence by occupation: New Zealand, 1972–1984. Int J Epidemiol.

[CR27] Froines JR, Hinds WC, Duffy RM, Lafuente EJ, Liu WC (1987). Exposure of firefighters to diesel emissions in fire stations. Am Ind Hyg Assoc J.

[CR28] Giles G, Staples M, Berry J (1993). Cancer incidence in Melbourne Metropolitan Fire Brigade members, 1980–1989. Health Rep.

[CR29] Glass DC, Pircher S, Del Monaco A, Hoorn SV, Sim MR (2016). Mortality and cancer incidence in a cohort of male paid Australian firefighters. Occup Environ Med.

[CR30] Glass DC, Del Monaco A, Pircher S, Vander Hoorn S, Sim MR (2017). Mortality and cancer incidence among male volunteer Australian firefighters. Occup Environ Med.

[CR31] Golka K, Weistenhöfer W (2008). Fire fighters, combustion products, and urothelial cancer. J Toxicol Environ Health B Crit Rev.

[CR32] Guidotti TL (1993). Mortality of urban firefighters in Alberta, 1927–1987. Am J Ind Med.

[CR33] Guidotti TL, Clough VM (1992). Occupational health concerns of firefighting. Annu Rev Public Health.

[CR34] Hansen ES (1990). A cohort study on the mortality of firefighters. Br J Ind Med.

[CR35] Hasenmeier P (2008) The History of Firefighter Personal Protective Equipment. https://www.fireengineering.com/articles/2008/06/the-history-of-firefighter-personal-protective-equipment.html. Accessed 09 Nov 2018

[CR36] Heyer N, Weiss NS, Demers P, Rosenstock L (1990). Cohort mortality study of Seattle fire fighters: 1945–1983. Am J Ind Med.

[CR37] Higgins JPT, Thompson SG (2002). Quantifying heterogeneity in a meta-analysis. Stat Med.

[CR38] Howe GR, Burch JD (1990). Fire fighters and risk of cancer: an assessment and overview of the epidemiologic evidence. Am J Epidemiol.

[CR39] International Agency for Research on Cancer (IARC) working group on the evaluation of carcinogenic risks to humans (2010). Painting, firefighting, and shiftwork. IARC Monogr Eval Carcinog Risks Hum.

[CR40] Jalilian H, Ziaei M, Weiderpass E, Rueegg CS, Khosravi Y, Kjaerheim K (2019). Cancer incidence and mortality among firefighters. Int J Cancer.

[CR41] Kullberg C, Andersson T, Gustavsson P, Selander J, Tornling G, Gustavsson A, Bigert C (2018). Cancer incidence in Stockholm firefighters 1958–2012: an updated cohort study. Int Arch Occup Environ Health.

[CR42] Lee W, Ahn Y-S, Lee S, Song BM, Hong S, Yoon J-H (2016). Occupational exposure to crystalline silica and gastric cancer: a systematic review and meta-analysis. Occup Environ Med.

[CR43] Leiter U, Garbe C (2008). Epidemiology of melanoma and nonmelanoma skin cancer–the role of sunlight. Adv Exp Med Biol.

[CR44] LeMasters GK, Genaidy AM, Succop P, Deddens J, Sobeih T, Barriera-Viruet H, Dunning K, Lockey J (2006). Cancer risk among firefighters: a review and meta-analysis of 32 studies. J Occup Environ Med.

[CR45] Lin L, Chu H (2018). Quantifying publication bias in meta-analysis. Biometrics.

[CR46] Ma F, Fleming LE, Lee DJ, Trapido E, Gerace TA, Lai H, Lai S (2005). Mortality in Florida professional firefighters, 1972 to 1999. Am J Ind Med.

[CR47] Ma F, Fleming LE, Lee DJ, Trapido E, Gerace TA (2006). Cancer incidence in Florida professional firefighters, 1981 to 1999. J Occup Environ Med.

[CR48] Manecksha RP, Fitzpatrick JM (2009). Epidemiology of testicular cancer. BJU Int.

[CR49] Mastromatteo E (1959). Mortality in city firemen, II. A study of mortality in firemen of a city fire department. AMA Arch Ind Health.

[CR50] Misner JE, Plowman SA, Boileau RA (1987). Performance differences between males and females on simulated firefighting tasks. J Occup Med.

[CR51] Moher D, Liberati A, Tetzlaff J, Altman DG (2009). Preferred reporting items for systematic reviews and meta-analyses: the PRISMA statement. J Clin Epidemiol.

[CR52] Morton W, Marjanovic D (1984). Leukemia incidence by occupation in the Portland-Vancouver metropolitan area. Am J Ind Med.

[CR53] Musk AW, Monson RR, Peters JM, Peters RK (1978). Mortality among Boston firefighters, 1915–1975. Br J Ind Med.

[CR54] National Cancer Institute (2018) SEER Stats Fact Sheets: Melanoma of the skin. https://seer.cancer.gov/statfacts/html/melan.html. Accessed 30 May 2019

[CR55] Paule RC, Mandel J (1982). Consensus values and weighting factors. J Res Natl Bur Stand.

[CR56] Pedersen JE, Petersen KU, Hansen J (2018). Historical changes in chemical exposures encountered by Danish firefighters. Scand J Work Environ Health.

[CR57] Petersen KKU, Pedersen JE, Bonde JP, Ebbehoej NE, Hansen J (2018). Long-term follow-up for cancer incidence in a cohort of Danish firefighters. Occup Environ Med.

[CR58] Pukkala E, Martinsen JI, Weiderpass E, Kjaerheim K, Lynge E, Tryggvadottir L, Sparén P, Demers PA (2014). Cancer incidence among firefighters: 45 years of follow-up in five Nordic countries. Occup Environ Med.

[CR59] Raj A, Mayberry JF, Podas T (2003). Occupation and gastric cancer. Postgrad Med J.

[CR60] Reisen F, Bhujel M, Leonard J (2014). Particle and volatile organic emissions from the combustion of a range of building and furnishing materials using a cone calorimeter. Fire Saf J.

[CR61] Rosénstock L, Demers P, Heyer NJ, Barnhart S (1990). Respiratory mortality among firefighters. Br J Ind Med.

[CR62] Sritharan J, Pahwa M, Demers PA, Harris SA, Cole DC, Parent M-E (2017). Prostate cancer in firefighting and police work: a systematic review and meta-analysis of epidemiologic studies. Environ Health.

[CR63] Tornling G, Gustavsson P, Hogstedt C (1994). Mortality and cancer incidence in Stockholm fire fighters. Am J Ind Med.

[CR64] Vena JE, Fiedler RC (1987). Mortality of a municipal-worker cohort: IV. Fire fighters. Am J Ind Med.

[CR65] Veroniki AA, Jackson D, Viechtbauer W, Bender R, Bowden J, Knapp G, Kuss O, Higgins JPT, Langan D, Salanti G (2016). Methods to estimate the between-study variance and its uncertainty in meta-analysis. Res Synth Methods.

[CR66] Welling R, Beaumont JJ, Petersen SJ, Alexeeff GV, Steinmaus C (2015). Chromium VI and stomach cancer: a meta-analysis of the current epidemiological evidence. Occup Environ Med.

[CR67] Youakim S (2006). Risk of cancer among firefighters: a quantitative review of selected malignancies. Arch Environ Occup Health.

[CR68] Youlden DR, Cramb SM, Baade PD (2008). The International epidemiology of lung cancer: geographical distribution and secular trends. J Thorac Oncol.

[CR69] Yung J, Li J, Jordan HT, Cone JE (2018). Prevalence of and factors associated with mammography and prostate-specific antigen screening among World Trade Center Health Registry enrollees, 2015–2016. Prev Med Rep.

[CR70] Zeig-Owens R, Webber MP, Hall CB, Schwartz T, Jaber N, Weakley J, Rohan TE, Cohen HW, Derman O, Aldrich TK, Kelly K, Prezant DJ (2011). Early assessment of cancer outcomes in New York City firefighters after the 9/11 attacks: an observational cohort study. Lancet.

