# Supplementary material for: Cancer risks of firefighters: a systematic review and meta-analysis of secular trends and region-specific differences
Source: Int Arch Occup Environ Health. 2020 Apr 18;93(7):839–52. doi: 10.1007/s00420-020-01539-0 (PMC7452930; doi:10.1007/s00420-020-01539-0)
Supplement: Supplementary file 1 — Supplementary file1 (DOCX 311 kb) [file 420_2020_1539_MOESM1_ESM.docx]

**Supplementary material**

**Cancer risk of firefighters: a systematic review and meta-analysis of secular trends and region-specific differences**

Swaantje Casjens, Thomas Brüning, Dirk Taeger

**Table S1** Search strategies in the PubMed database

| PubMed | ((((((((((((((("fire fighter"[All Fields] OR "fire fighters"[All Fields]) OR ("firefighters"[MeSH Terms] OR "firefighters"[All Fields] OR "firefighter"[All Fields])) OR ("firefighters"[MeSH Terms] OR "firefighters"[All Fields] OR "fireman"[All Fields])) OR firemen[All Fields]) OR firefighting[All Fields]) OR "fire fighting"[All Fields]) OR "fire Brigade"[All Fields]) OR "fire Brigades"[All Fields]) OR "fire service"[All Fields]) OR "fire services"[All Fields]) OR "fire department"[All Fields]) OR "fire departments"[All Fields]) OR "fire station"[All Fields]) OR "fire stations"[All Fields]) AND "humans"[MeSH Terms]) AND (((((("neoplasms"[MeSH Terms] OR "neoplasms"[All Fields] OR "cancer"[All Fields]) OR ("neoplasms"[MeSH Terms] OR "neoplasms"[All Fields] OR "neoplasm"[All Fields])) OR ("mortality"[Subheading] OR "mortality"[All Fields] OR "mortality"[MeSH Terms])) OR ("neoplasm metastasis"[MeSH Terms] OR ("neoplasm"[All Fields] AND "metastasis"[All Fields]) OR "neoplasm metastasis"[All Fields] OR "metastasis"[All Fields])) OR ("tumour"[All Fields] OR "neoplasms"[MeSH Terms] OR "neoplasms"[All Fields] OR "tumor"[All Fields])) OR ("carcinoma"[MeSH Terms] OR "carcinoma"[All Fields])) AND (("0001/01/01"[PDAT] : "2019/01/01"[PDAT]) AND "humans"[MeSH Terms] AND English[lang]) |
| --- | --- |

**Table S2** Extracted estimates on cancer incidence and mortality from 25 cohort studies

| Reference | International classification of disease code (ICD) - 10 | | | | | | | | | | | | | | | | | | | | | | | | | | | | | | | | | | | | | | | | | | | | | | | | |
| --- | --- | --- | --- | --- | --- | --- | --- | --- | --- | --- | --- | --- | --- | --- | --- | --- | --- | --- | --- | --- | --- | --- | --- | --- | --- | --- | --- | --- | --- | --- | --- | --- | --- | --- | --- | --- | --- | --- | --- | --- | --- | --- | --- | --- | --- | --- | --- | --- | --- |
|  | C00-C97 | C00-C14 | C00 | C15-C26 | C15 | C16 | C17 | C18 | C18-C20 | C18-C21 | C19-C21 | C20 | C22-C24 | C22 | C23, C24 | C25 | C30-C39 | C32 | C33, C34 | C34 | C40, C41 | C43, C44 | | C43 | C44 | C45 | C48, C49 | C50 | C60-C68 | C60-C63 | C61 | C62 | C64-C68 | C64 | C64, C65 | C64-C66 | C66-C68 | C67 | C67, C68 | C69 | C70-C72 | C71 | C73 | C81-C96 | C81 | C82-C85 | C83 | C90 | C91-C95 |
| **Cancer incidence** | | | | | | | | | | | | | | | | | | | | | | | | | | | | | | | | | | | | | | | | | | | | | | | | | |
| Ahn et al. 2012 | + |  |  |  | + | + | + |  | + |  |  |  | + | + | + | + |  | + | + |  | + |  | |  |  |  |  |  |  |  | + |  |  | + |  |  |  | + |  |  | + |  | + | + |  | + |  |  | + |
| Bates et al. 2001 | + |  |  |  | + | + |  | + |  |  | + |  |  |  |  | + |  |  | + |  |  |  | | + |  |  |  |  |  |  | + | + |  |  |  | + |  | + |  |  | + |  |  |  |  |  |  |  | + |
| Daniels et al. 2014 | + |  |  |  | + | + |  |  |  |  | + |  |  |  |  |  |  |  | + |  |  |  | |  |  |  |  | + | + | + | + |  | + |  |  | + |  |  |  | + |  |  |  |  |  |  |  |  | + |
| Demers et al. 1994 | + | + |  |  | + | + |  | + |  | + | + |  |  |  |  | + |  | + | + |  |  |  | | + |  |  |  | + |  |  | + |  | + |  |  | + |  | + |  |  | + |  | + |  | + | + |  | + | + |
| Glass et al. 2016 | + | + |  |  | + | + |  | + |  | + |  | + |  | + |  | + |  | + | + |  |  |  | | + |  | + |  | + | + | + | + | + | + | + |  |  |  | + |  |  | + | + | + | + | + | + |  | + | + |
| Kullberg et al. 2018 | + |  | + |  | + | + |  | + |  |  | + |  | + |  |  | + |  |  | + |  |  | + | | + | + |  | + |  |  |  | + |  |  |  |  | + | + |  |  |  | + |  |  | + | + |  |  | + | + |
| Ma et al. 2006 | + | + |  |  | + | + |  | + |  |  |  | + |  | + |  | + |  | + |  | + | + |  | |  | + |  |  | + |  |  | + | + |  |  | + |  |  | + |  | + |  | + | + | + | + | + |  |  | + |
| Morton and Marjanovic 1984 |  |  |  |  |  |  |  |  |  |  |  |  |  |  |  |  |  |  |  |  |  |  | |  |  |  |  |  |  |  |  |  |  |  |  |  |  |  |  |  |  |  |  |  |  |  |  |  | + |
| Petersen et al. 2018a | + |  |  |  |  |  |  |  | + |  |  | + |  |  |  | + |  |  |  |  |  | + | | + | + |  |  |  |  |  | + | + |  |  |  |  |  | + |  |  |  |  |  |  |  | + |  |  |  |
| Pukkala et al. 2014 | + | + | + |  | + | + | + | + |  | + | + |  | + | + | + | + |  | + | + |  |  | + | | + | + | + | + |  |  |  | + | + |  |  | + |  | + |  |  |  |  | + | + |  |  |  |  | + | + |
| **Cancer mortality** | | | | | | | | | | | | | | | | | | | | | | | | | | | | | | | | | | | | | | | | | | | | | | | | | |
| Ahn and Jeong 2015 | + |  |  |  |  | + |  |  | + |  |  |  |  | + |  |  |  |  |  | + |  |  |  | |  |  |  |  |  |  |  |  |  |  |  |  |  |  |  |  |  |  |  | + |  |  | + |  | + |
| Amadeo et al. 2015 | + | + |  |  | + | + |  | + |  |  |  |  |  | + |  | + |  |  |  | + |  | + |  | |  |  |  | + |  |  | + |  |  |  |  |  |  | + |  |  |  |  |  | + |  |  |  |  |  |
| Aronson et al. 1994 | + |  |  |  | + | + |  | + |  |  | + |  |  | + |  | + |  | + | + |  |  |  | + | |  |  |  |  |  |  | + | + |  |  |  | + |  | + |  |  | + |  |  |  | + |  |  | + |  |
| Baris et al. 2001 | + | + |  |  | + | + |  | + |  |  | + |  | + |  |  | + |  | + | + |  |  | + |  | |  |  |  |  |  |  | + |  |  |  |  | + |  | + |  |  | + |  |  |  |  | + |  | + | + |
| Bates et al. 2001 | + |  |  |  |  |  |  | + |  |  | + |  |  |  |  |  |  |  | + |  |  |  | + | |  |  |  |  |  |  |  |  |  |  |  |  |  | + |  |  | + |  |  | + |  |  |  |  |  |
| Berg and Howell 1975 |  |  |  |  |  |  |  |  | + |  |  |  |  |  |  |  |  |  |  |  |  |  |  | |  |  |  |  |  |  |  |  |  |  |  |  |  |  |  |  |  |  |  |  |  |  |  |  |  |
| Daniels et al. 2014 | + |  |  |  | + | + |  |  |  |  | + |  |  |  |  |  |  |  | + |  |  |  |  | |  |  |  | + | + |  | + | + | + |  |  | + |  |  | + |  |  |  |  |  |  |  |  |  | + |
| Demers et al. 1992 | + | + |  |  | + | + |  | + |  | + | + |  | + |  |  | + |  | + | + |  |  | + |  | |  |  |  |  |  |  | + |  | + |  |  | + |  |  | + |  | + |  |  |  | + |  | + |  | + |
| Deschamps et al. 1995 | + | + |  | + |  |  |  |  |  |  |  |  |  |  |  |  | + |  |  |  |  |  |  | |  |  |  |  | + |  |  |  |  |  |  |  |  |  |  |  |  |  |  |  |  |  |  |  |  |
| Eliopulos et al. 1984 | + |  |  |  |  |  |  |  |  |  |  |  |  |  |  |  | + |  |  |  |  |  |  | |  |  |  |  |  |  |  |  |  |  |  |  |  |  |  |  |  |  |  |  |  |  |  |  |  |
| Glass et al. 2016 | + |  |  |  |  |  |  |  |  |  |  |  |  |  |  |  |  |  |  |  |  |  |  | |  |  |  |  |  |  |  |  |  |  |  |  |  |  |  |  |  |  |  |  |  |  |  |  |  |
| Guidotti 1993 | + | + |  |  |  | + |  |  |  | + |  |  |  |  |  | + |  |  | + |  |  | + |  | |  |  |  |  |  |  | + |  |  |  |  | + |  | + |  |  |  | + |  |  |  |  |  |  |  |
| Hansen 1990 | + |  |  |  |  |  |  |  |  |  |  |  |  |  |  |  |  |  | + |  |  |  |  | |  |  |  |  |  |  |  |  |  |  |  |  |  |  |  |  |  |  |  |  |  |  |  |  |  |
| Ma et al. 2005 | + | + |  | + | + | + |  | + |  |  |  | + |  | + |  | + |  |  | + |  |  | + |  | | + |  |  | + |  |  | + |  |  |  |  |  |  | + |  |  |  | + |  | + | + | + |  |  | + |
| Mastromatteo 1959 | + |  |  |  |  |  |  |  |  |  |  |  |  |  |  |  |  |  |  |  |  |  |  | |  |  |  |  |  |  |  |  |  |  |  |  |  |  |  |  |  |  |  |  |  |  |  |  |  |
| Musk et al. 1978 | + |  |  | + |  |  |  |  |  |  |  |  |  |  |  |  | + |  |  |  |  |  |  | |  |  |  |  | + |  |  |  |  |  |  |  |  |  |  |  |  | + |  | + |  |  |  |  |  |
| Tornling et al. 1994 | + |  |  |  |  | + |  | + |  |  | + |  |  | + |  | + |  |  | + |  |  |  |  | |  |  |  |  |  |  | + |  |  |  |  | + |  |  |  |  |  | + |  | + |  |  |  |  |  |
| Vena and Fiedler 1987 | + |  |  | + | + | + |  | + |  |  |  | + | + |  |  | + | + |  |  |  |  |  |  | |  |  |  |  |  |  | + |  |  |  |  | + |  | + |  |  |  | + |  | + |  |  |  |  |  |

**Table S3** Meta-relative risk estimates (mRR) and 95% confidence intervals (CI) for cancer incidence and mortality of single ICD-10 codes of specific sites presented as combined estimates in Tables 2 and 3

| Disease (ICD-10) | # Studies | Study IDs | mRR (95% CI) | I^2^ (p value) | τ² |
| --- | --- | --- | --- | --- | --- |
| **Cancer incidence** |  |  |  |  |  |
| Colorectal combined (C18-C21) | 5 | 1,10,13,22,23 | 1.08 (1.00 - 1.16) | 0 (0.539) | 0 |
| Colorectal (C18-C20) | 2 | 1,22 | 1.10 (0.77 - 1.42) | 67.5 (0.080) | 0.040 |
| Colorectal (C18-C21) | 3 | 10 ,13 ,23 | 1.08 (0.99 - 1.17) | 0 (0.988) | 0 |
| Rectum combined (C19-C21) | 8 | 6,8,10,13,16,18,22,23 | 1.09 (0.99 - 1.19) | 0 (0.819) | 0 |
| Rectum (C19-C21) | 5 | 6,8,10,16,23 | 1.09 (0.97 - 1.20) | 0 (0.732) | 0 |
| Rectum (C20) | 3 | 13,18,22 | 1.10 (0.91 - 1.29) | 0 (0.444) | 0 |
| Trachea and lung combined (C33-C34) | 8 | 1,6,8,10,13,16,18,23 | 0.91 (0.78 - 1.03) | 87.7 (<0.001) | 0 |
| Trachea and lung (C33, C34) | 7 | 1,6,8,10,13,16,23 | 0.97 (0.85 - 1.08) | 71.1 (0.002) | 0.011 |
| Lung (C34) | 1 | 18 | 0.65 (0.54 - 0.78) | - | - |
| Skin combined (C43-C44) | 7 | 6,10,13,16,18,22,23 | 1.16 (0.98 - 1.35) | 74.2 (0.001) | 0.042 |
| Skin (C43, C44) | 3 | 16,22,23 | 1.02 (0.67 - 1.36) | 85.6 (0.001) | 0.079 |
| Kidney combined (C64-C66) | 8 | 1,6,8,10,13,16,18,23 | 0.98 (0.75 - 1.20) | 62.6 (0.009) | 0.053 |
| Kidney (C64) | 2 | 1,13 | 1.22 (0.65 - 1.80) | 53.0 (0.145) | 0.120 |
| Kidney (C64, C65) | 2 | 18,23 | 0.90 (0.72 - 1.07) | 0 (0.351) | 0 |
| Kidney (C64-C66) | 4 | 6,8,10,16 | 0.89 (0.49 - 1.29) | 74.8 (0.008) | 0.080 |
| Bladder combined (C67-C68) | 7 | 1,6,8,10,13,18,22 | **1.14 (1.04 - 1.23)** | 0 (0.592) | 0 |
| Bladder (C66-C68) | 2 | 16,23 | 0.97 (0.60 - 1.33) | 73.1 (0.054) | 0.051 |
| Bladder (C67) | 6 | 1,6,10,13,18,22 | **1.18 (1.01 - 1.34)** | 0 (0.492) | 0.005 |
| Bladder (C67-C68) | 1 | 8 | 1.11 (0.99 - 1.24) | - | - |
| Brain combined (C70-C72) | 7 | 1,6,10,13,16,18,23 | **0.81 (0.65 - 0.98)** | 6.2 (0.380) | 0 |
| Brain (C70-C72) | 5 | 1,6,10,13,16 | 0.87 (0.57 - 1.16) | 0 (0.489) | 0 |
| Brain (C71) | 3 | 13,18,23 | 0.78 (0.56 - 1.00) | 35.5 (0.212) | 0 |
| Non-Hodgkin lymphoma combined (C82-C85) | 6 | 1,10,13,16,18,22 | 1.05 (0.83 - 1.28) | 0 (0.484) | 0.018 |
| Non-Hodgkin lymphoma (C82-C85) | 5 | 1,10,13,18,22 | 1.09 (0.86 - 1.32) | 0 (0.577) | 0.014 |
| Non-Hodgkin lymphoma (C83) | 1 | 16 | 0.68 (0.25 - 1.48) | - | - |
|  |  |  |  |  |  |
| **Cancer mortality** |  |  |  |  |  |
| Colorectal combined (C18-C21) | 4 | 2,7,9,14 | 1.47 (0.52 - 2.42) | 86.5 (<0.001) | 0.869 |
| Colorectal (C18-C20) | 2 | 2,7 | 1.72 (0 - 3.81) | 94.7 (<0.001) | 2.227 |
| Colorectal (C18-C21) | 2 | 9,14 | 1.18 (0.47 - 1.90) | 63.0 (0.100) | 0.204 |
| Rectum combined (C19-C21) | 9 | 3,4,5,6,8,9,17,24,25 | **1.35 (1.12 - 1.59)** | 0 (0.692) | 0.029 |
| Rectum (C19-C20) | 1 | 3 | **1.36 (0.86 - 2.04)** | - | - |
| Rectum (C19-C21) | 6 | 4,5,6,8,9,24 | **1.36 (1.07 - 1.65)** | 0 (0.553) | 0.038 |
| Rectum (C20) | 2 | 17,25 | 1.44 (0.33 - 2.55) | 34.3 (0.217) | 0.436 |
| Trachea and lung combined (C33-C34) | 11 | 2,3,4,5,6,8,9,14,15,17,24 | 0.98 (0.86 - 1.11) | 72.1 (<0.001) | 0.025 |
| Trachea and lung (C33, C34) | 9 | 4,5,6,8,9,14,15,17,24 | 1.05 (0.96 - 1.14) | 27.4 (0.201) | 0.006 |
| Lung (C34) | 2 | 2,3 | 0.75 (0.48 - 1.02) | 82.8 (0.016) | 0.026 |
| Skin combined (C43-C44) | 7 | 3,4,5,6,9,14,17 | 0.87 (0.59 - 1.15) | 0 (0.927) | 0 |
| Skin (C43, C44) | 5 | 3,5,9,14,17 | 0.89 (0.59 - 1.19) | 0 (0.823) | 0 |
| Malignant melanoma of skin (C43) | 2 | 4,6 | 0.69 (0 - 1.50) | 0 (0.888) | 0 |
| Other malignant skin neoplasms (C44) | 1 | 17 | 0.89 (0.52 - 1.42) | - | - |
| Kidney combined (C64-C66) | 8 | **3**,4,5,8,9,14,24,25 | 1.18 (0.42 - 1.94) | 76.1 (<0.001) | 1.008 |
| Kidney (C64) | 1 | 3 | 0.63 (0.30 - 1.16) | - | - |
| Kidney (C64-C66) | 7 | 4,5,8,9,14,24,25 | 1.28 (0.40 - 2.15) | 75.8 (<0.001) | 1.174 |
| Bladder combined (C67-C68) | 9 | 3,4,5,6,8,9,14,17,25 | 1.44 (0.82 - 2.06) | 74.4 (<0.001) | 0.673 |
| Bladder (C67) | 7 | 3,4,5,6,14,17,25 | **1.72 (1.05 - 2.38)** | 45.6 (0.088) | 0.528 |
| Bladder (C67, C68) | 2 | 8,9 | 0.67 (0.00 - 1.37) | 93.9 (<0.001) | 0.203 |
| Brain combined (C70-C72) | 9 | 4,5,6,9,14,17,21,24,25 | 1.42 (0.90 - 1.93) | 61.3 (0.008) | 0.418 |
| Brain (C70-C72) | 4 | 4,5,6,9 | 1.37 (0.57 - 2.16) | 75.3 (0.007) | 0.501 |
| Brain (C71) | 5 | 14,17,21,24,25 | 1.48 (0.71 - 2.26) | 48.2 (0.103) | 0.507 |
| Non-Hodgkin lymphoma combined (C82-C85) | 4 | 4,5,9,17 | 1.31 (0.92 - 1.70) | 0 (0.446) | 0 |
| Non-Hodgkin lymphoma (C82-C85) | 2 | 5,17 | 1.13 (0.41 - 1.85) | 53.1 (0.144) | 0.145 |
| Non-Hodgkin lymphoma (C83) | 2 | 4,9 | 1.56 (0.79 - 2.34) | 0 (0.699) | 0 |
| Leukemia combined (C91-C95) | 6 | 2,4,5,8,9,17 | 1.04 (0.88 - 1.19) | 0 (0.459) | 0 |
| Leukemia (C91-C95) | 5 | 2,5,8,9,17 | 1.03 (0.88 - 1.18) | 7.4 (0.365) | 0 |
| Lymphoid and myeloid leukemia (C91-C92) | 1 | 4 | 1.48 (0.64 - 2.91) | - | - |

*Study IDs* IDs of included studies in this meta-analysis as depicted in Table 1, *mRR* meta-relative risk assessed with inverse-variance random effects meta-analysis and Paule-Mandel heterogeneity variance estimator τ², *p value* p value of heterogeneity test

**Table S4** Meta-relative risk estimates (mRR) and 95% confidence intervals (CI) assessed with random effects models and Paule-Mandel heterogeneity variance estimator (τ²) stratified by period of employment

| Disease (ICD-10 code) | Employment start | # Studies | Study IDs | mRR | 95% CI | | I^2^ (p value) | τ² |
| --- | --- | --- | --- | --- | --- | --- | --- | --- |
| **Cancer incidence** |  |  |  |  |  |  |  |  |
| All cancer (C00-C97) | Overall | 9 | 1,6,8,10,13,16,18,22,23 | 1.00 | 0.93 | 1.07 | 91.3 (<0.001) | 0.010 |
|  | < 1950 | 2 | 10,16 | 0.95 | 0.67 | 1.24 | 91.8 (<0.001) | 0.038 |
|  | 1950 – ‘70 | 3 | 8, 22,23 | **1.08** | **1.05** | **1.11** | 13.6 (0.314) | 0 |
|  | > 1970 | 4 | 1,6,13,18 | 0.96 | 0.86 | 1.06 | 91.7 (<0.001) | 0.009 |
| Buccal cavity and pharynx (C00-C14) | Overall | 4 | 10,13,18,23 | 0.87 | 0.72 | 1.02 | 41.8 (0.161) | 0.003 |
|  | < 1950 | 1 | 10 | 1.10 | 0.60 | 2.00 | - | - |
|  | 1950 – ‘70 | 1 | 23 | 0.92 | 0.62 | 1.31 | - | - |
|  | > 1970 | 2 | 13,18 | 0.81 | 0.54 | 1.08 | 72.0 (0.059) | 0.022 |
| Esophagus (C15) | Overall | 8 | 1,6,8,10,13,16,18,23 | 1.06 | 0.76 | 1.36 | 65.7 (0.005) | 0.088 |
|  | < 1950 | 2 | 10,16 | 1.11 | 0.42 | 1.79 | 0 (0.705) | 0 |
|  | 1950 – ‘70 | 2 | 8,23 | 1.35 | 0.64 | 2.05 | 83.4 (0.014) | 0.234 |
|  | > 1970 | 4 | 1,6,13,18 | 0.74 | 0.44 | 1.04 | 0 (0.625) | 0 |
| Stomach (C16) | Overall | 8 | 1,6,8,10,13,16,18,23 | 1.08 | 0.80 | 1.35 | 71.1 (0.001) | 0.109 |
|  | < 1950 | 2 | 10,16 | **1.75** | **1.31** | **2.19** | 0 (0.541) | 0 |
|  | 1950 – ‘70 | 2 | 8,23 | 1.13 | 0.99 | 1.27 | 0 (0.556) | 0 |
|  | > 1970 | 4 | 1,6,13,18 | 0.83 | 0.62 | 1.05 | 68.2 (0.024) | 0.015 |
| Colon (C18) | Overall | 6 | 6,10,13,16,18,23 | **1.11** | **1.00** | **1.21** | 19.6 (0.285) | 0 |
|  | < 1950 | 2 | 10,16 | 0.97 | 0.68 | 1.27 | 0 (0.413) | 0 |
|  | 1950 – ‘70 | 1 | 23 | 1.14 | 0.99 | 1.31 | - | - |
|  | > 1970 | 3 | 6,13,18 | 1.05 | 0.76 | 1.34 | 56.4 (0.101) | 0.038 |
| Colorectal combined (C18-C21) | Overall | 5 | 1,10,13,22,23 | **1.08** | **1.00** | **1.16** | 0 (0.539) | 0 |
|  | < 1950 | 1 | 10 | 1.06 | 0.74 | 1.48 | - | - |
|  | 1950 – ‘70 | 2 | 22,23 | 1.04 | 0.92 | 1.16 | 18.4 (0.268) | 0.002 |
|  | > 1970 | 2 | 1,13 | 1.15 | 0.98 | 1.32 | 0 (0.329) | 0.004 |
| Rectum combined (C19-C21) | Overall | 8 | 6,8,10,13,16,18,22,23 | 1.09 | 0.99 | 1.19 | 0 (0.819) | 0 |
|  | < 1950 | 2 | 10,16 | 1.14 | 0.75 | 1.52 | 0 (0.567) | 0 |
|  | 1950 – ‘70 | 3 | 8,22,23 | 1.09 | 0.97 | 1.21 | 0 (0.413) | 0 |
|  | > 1970 | 3 | 6,13,18 | 1.08 | 0.86 | 1.30 | 0 (0.474) | 0 |
| Rectum (C19-C21) | Overall | 5 | 6,8,10,16,23 | 1.09 | 0.97 | 1.20 | 0 (0.732) | 0 |
|  | < 1950 | 2 | 10,16 | 1.14 | 0.75 | 1.52 | 0 (0.567) | 0 |
|  | 1950 – ‘70 | 2 | 8,23 | 1.08 | 0.91 | 1.24 | 37.9 (0.204) | 0.007 |
|  | > 1970 | 1 | 6 | 1.15 | 0.50 | 2.20 | - | - |
| Rectum (C20) | Overall | 3 | 13,18,22 | 1.10 | 0.91 | 1.29 | 0 (0.444) | 0 |
|  | < 1950 | 0 | - | - | - | - | - | - |
|  | 1950 – ‘70 | 1 | 22 | 1.16 | 0.84 | 1.60 | - | - |
|  | > 1970 | 2 | 13,18 | 1.06 | 0.77 | 1.35 | 31.0 (0.229) | 0.015 |
| Liver (C22) | Overall | 4 | 1,13,18,23 | **0.81** | **0.65** | **0.98** | 16.6 (0.309) | 0 |
|  | < 1950 | 0 | - | - | - | - | - | - |
|  | 1950 – ‘70 | 1 | 23 | 0.91 | 0.59 | 1.34 | - | - |
|  | > 1970 | 3 | 1,13,18 | **0.79** | **0.60** | **0.97** | 34.5 (0.217) | 0 |
| Pancreas (C25) | Overall | 8 | 1,6,10,13,16,18,22,23 | 1.08 | 0.88 | 1.28 | 39.8 (0.114) | 0.021 |
|  | < 1950 | 2 | 10,16 | 1.08 | 0.57 | 1.58 | 0 (0.915) | 0 |
|  | 1950 – ‘70 | 2 | 22,23 | 1.30 | 0.95 | 1.64 | 6.95 (0.300) | 0.033 |
|  | > 1970 | 4 | 1,6,13,18 | 0.86 | 0.59 | 1.14 | 40.1 (0.171) | 0.003 |
| Larynx (C32) | Overall | 5 | 1,10,13,18,23 | 0.88 | 0.66 | 1.10 | 0 (0.550) | 0 |
|  | < 1950 | 1 | 10 | 1.00 | 0.30 | 2.30 | - | - |
|  | 1950 – ‘70 | 1 | 23 | 1.06 | 0.72 | 1.50 | - | - |
|  | > 1970 | 3 | 1,13,18 | 0.75 | 0.46 | 1.04 | 0 (0.721) | 0 |
| Trachea and lung combined (C33-C34) | Overall | 8 | 1,6,8,10,13,16,18,23 | 0.91 | 0.78 | 1.03 | 87.7 (<0.001) | 0 |
|  | < 1950 | 2 | 10,16 | 0.91 | 0.69 | 1.13 | 10.3 (0.291) | 0 |
|  | 1950 – ‘70 | 2 | 8,23 | 1.06 | 0.89 | 1.24 | 83.5 (0.014) | 0.014 |
|  | > 1970 | 4 | 1,6,13,18 | **0.76** | **0.62** | **0.91** | 57.2 (0.072) | 0.008 |
| Trachea and lung (C33, C34) | Overall | 7 | 1,6,8,10,13,16,23 | 0.97 | 0.85 | 1.08 | 71.1 (0.002) | 0.011 |
|  | < 1950 | 2 | 10,16 | 0.91 | 0.69 | 1.13 | 10.3 (0.291) | 0 |
|  | 1950 – ‘70 | 2 | 8,23 | 1.06 | 0.89 | 1.24 | 83.5 (0.014) | 0.014 |
|  | > 1970 | 3 | 1,6,13 | **0.83** | **0.68** | **0.98** | 0 (0.417) | 0 |
| Skin combined (C43, C44) | Overall | 7 | 6,10,13,16,18,22,23 | 1.16 | 0.98 | 1.35 | 74.2 (0.001) | 0.042 |
|  | < 1950 | 2 | 10,16 | 0.84 | 0.35 | 1.33 | 52.9 (0.145) | 0.059 |
|  | 1950 – ‘70 | 2 | 22,23 | 1.15 | 0.87 | 1.42 | 83.5 (0.014) | 0.035 |
|  | > 1970 | 3 | 6,13,18 | **1.32** | **1.14** | **1.50** | 20.7 (0.283) | 0.011 |
| Skin (C43, C44) | Overall | 3 | 16,22,23 | 1.02 | 0.67 | 1.36 | 85.6 (0.001) | 0.079 |
|  | < 1950 | 1 | 16 | 0.66 | 0.41 | 1.03 | - | - |
|  | 1950 – ‘70 | 2 | 22,23 | 1.15 | 0.87 | 1.42 | 83.5 (0.014) | 0.035 |
|  | > 1970 | 0 | - | - | - | - | - | - |
| Malignant melanoma of skin (C43) | Overall | 6 | 6,10,13,16,22,23 | 1.19 | 0.89 | 1.48 | 78.8 (<0.001) | 0.090 |
|  | < 1950 | 2 | 10,16 | 0.73 | 0 | 1.61 | 89.1 (0.002) | 0.287 |
|  | 1950 – ‘70 | 2 | 22,23 | **1.26** | **1.08** | **1.44** | 0 (0.909) | 0 |
|  | > 1970 | 2 | 6,13 | **1.43** | **1.27** | **1.58** | 0 (0.766) | 0 |
| Other malignant neoplasms of the skin (C44) | Overall | 4 | 16,18,22,23 | 1.10 | 0.90 | 1.30 | 63.2 (0.043) | 0.026 |
|  | < 1950 | 1 | 16 | 0.85 | 0.49 | 1.35 | - | - |
|  | 1950 – ‘70 | 2 | 22,23 | 1.14 | 0.77 | 1.50 | 84.4 (0.011) | 0.060 |
|  | > 1970 | 1 | 18 | 1.17 | 0.95 | 1.42 | - | - |
| Mesothelioma (C45) | Overall | 2 | 13,23 | **1.46** | **1.01** | **1.90** | 0 (0.739) | 0 |
|  | < 1950 | 0 | - | - | - | - | - | - |
|  | 1950 – ‘70 | 1 | 23 | 1.55 | 0.90 | 2.48 | - | - |
|  | > 1970 | 1 | 13 | 1.33 | 0.66 | 2.37 | - | - |
| Breast (C50) | Overall | 4 | 8,10,13,18 | 1.23 | 0.27 | 2.19 | 28.6 (0.24) | 0.513 |
|  | < 1950 | 1 | 10 | 2.40 | 0.10 | 13.3 | - | - |
|  | 1950 – ‘70 | 1 | 8 | 0.79 | 0.29 | 1.72 | - | - |
|  | > 1970 | 2 | 13,18 | 1.44 | 0 | 3.38 | 74.2 (0.049) | 1.584 |
| Prostate (C61) | Overall | 9 | 1,6,8,10,13,16,18,22,23 | 1.10 | 0.97 | 1.22 | 75.0 (<0.001) | 0.025 |
|  | < 1950 | 2 | 10,16 | 1.03 | 0.33 | 1.74 | 94.0 (<0.001) | 0.243 |
|  | 1950 – ‘70 | 3 | 8, 22,23 | **1.08** | **1.00** | **1.15** | 54.9 (0.109) | 0.002 |
|  | > 1970 | 4 | 1,6,13,18 | **1.18** | **1.09** | **1.27** | 0 (0.672) | 0 |
| Testis (C62) | Overall | 5 | 6,13,18,22,23 | 1.26 | 0.87 | 1.65 | 77.0 (0.002) | 0.141 |
|  | < 1950 | 0 | - | - | - | - | - | - |
|  | 1950 – ‘70 | 2 | 22,23 | 0.87 | 0.17 | 1.58 | 86.4 (0.007) | 0.204 |
|  | > 1970 | 3 | 6,13,18 | **1.54** | **1.29** | **1.79** | 0 (0.929) | 0 |
| Kidney combined (C64-C66) | Overall | 8 | 1,6,8,10,13,16,18,23 | 0.98 | 0.75 | 1.20 | 62.6 (0.009) | 0.053 |
|  | < 1950 | 2 | 10,16 | 0.55 | 0.06 | 1.03 | 0 (0.793) | 0 |
|  | 1950 – ‘70 | 2 | 8,23 | 1.10 | 0.79 | 1.42 | 77.4 (0.036) | 0.041 |
|  | > 1970 | 4 | 1,6,13,18 | 1.00 | 0.63 | 1.37 | 45.4 (0.139) | 0.077 |
| Kidney (C64-C66) | Overall | 4 | 6,8,10,16 | 0.89 | 0.49 | 1.29 | 74.8 (0.008) | 0.080 |
|  | < 1950 | 2 | 10,16 | 0.55 | 0.06 | 1.03 | 0 (0.793) | 0 |
|  | 1950 – ‘70 | 1 | 8 | **1.26** | **1.06** | **1.47** | - | - |
|  | > 1970 | 1 | 6 | 0.57 | 0.10 | 2.10 | - | - |
| Bladder combined (C67-C68) | Overall | 7 | 1,6,8,10,13,18,22 | **1.14** | **1.04** | **1.23** | 0 (0.592) | 0 |
|  | < 1950 | 1 | 10 | 1.20 | 0.70 | 1.90 | - | - |
|  | 1950 – ‘70 | 2 | 8,22 | **1.11** | **1.01** | **1.22** | 0 (0.860) | 0 |
|  | > 1970 | 4 | 6,8,10,16 | 1.19 | 0.89 | 1.49 | 31.7 (0.222) | 0.038 |
| Bladder (C67) | Overall | 6 | 1,6,10,13,18,22 | **1.18** | **1.01** | **1.34** | 0 (0.492) | 0.005 |
|  | < 1950 | 1 | 10 | 1.20 | 0.70 | 1.90 | - | - |
|  | 1950 – ‘70 | 1 | 22 | 1.14 | 0.89 | 1.48 | - | - |
|  | > 1970 | 4 | 1,6,13,18 | 1.19 | 0.89 | 1.49 | 31.7 (0.222) | 0.038 |
| Brain combined (C70-C72) | Overall | 7 | 1,6,10,13,16,18,23 | **0.81** | **0.65** | **0.98** | 6.2 (0.380) | 0 |
|  | < 1950 | 2 | 10,16 | 1.14 | 0.53 | 1.74 | 0 (0.934) | 0 |
|  | 1950 – ‘70 | 1 | 23 | 0.86 | 0.66 | 1.10 | - | - |
|  | > 1970 | 4 | 1,6,13,18 | **0.70** | **0.44** | **0.95** | 1.9 (0.383) | 0 |
| Brain (C70-C72) | Overall | 5 | 1,6,10,13,16 | 0.87 | 0.57 | 1.16 | 0 (0.489) | 0 |
|  | < 1950 | 2 | 10,16 | 1.14 | 0.53 | 1.74 | 0 (0.934) | 0 |
|  | 1950 – ‘70 | 0 | - | - | - | - | - | - |
|  | > 1970 | 3 | 1,6,13 | 0.78 | 0.44 | 1.12 | 2.60 (0.358) | 0 |
| Brain (C71) | Overall | 3 | 13,18,23 | **0.78** | **0.56** | **1.00** | 35.5 (0.212) | 0 |
|  | < 1950 | 0 | - | - | - | - | - | - |
|  | 1950 – ‘70 | 1 | 23 | 0.92 | 0.64 | 1.30 | - | - |
|  | > 1970 | 2 | 13,18 | **0.66** | **0.37** | **0.96** | 0 (0.364) | 0 |
| Thyroid (C73) | Overall | 5 | 1,10,13,18,23 | 1.26 | 0.98 | 1.54 | 0 (0.623) | 0.011 |
|  | < 1950 | 1 | 10 | 0.80 | 0.20 | 4.20 | - | - |
|  | 1950 – ‘70 | 1 | 23 | 1.28 | 0.75 | 2.05 | - | - |
|  | > 1970 | 3 | 1,13,18 | 1.29 | 0.84 | 1.75 | 15.4 (0.307) | 0.086 |
| Lymphohematopoietic (C81-C96) | Overall | 4 | 1,13,16,18 | 0.90 | 0.63 | 1.17 | 76.1 (0.006) | 0.055 |
|  | < 1950 | 1 | 16 | 0.73 | 0.43 | 1.16 | - | - |
|  | 1950 – ‘70 | 0 | - | - | - | - | - | - |
|  | > 1970 | 3 | 1,13,18 | 0.95 | 0.60 | 1.31 | 83.5 (0.002) | 0.080 |
| Hodgkin's disease (C81) | Overall | 4 | 10,13,16,18 | 0.84 | 0.44 | 1.24 | 0 (0.906) | 0 |
|  | < 1950 | 2 | 10,16 | 1.05 | 0 | 2.22 | 0 (0.555) | 0 |
|  | 1950 – ‘70 | 0 | - | - | - | - | - | - |
|  | > 1970 | 2 | 13,18 | 0.81 | 0.38 | 1.24 | 0 (0.723) | 0 |
| Non-Hodgkin lymphoma combined (C82-C85) | Overall | 6 | 1,10,13,16,18,22 | 1.05 | 0.83 | 1.28 | 0 (0.484) | 0.018 |
|  | < 1950 | 2 | 10,16 | 0.78 | 0.30 | 1.27 | 0 (0.567) | 0 |
|  | 1950 – ‘70 | 1 | 22 | 1.02 | 0.68 | 1.53 | - | - |
|  | > 1970 | 3 | 1,13,18 | 1.19 | 0.79 | 1.60 | 23.5 (0.270) | 0.076 |
| Non-Hodgkin lymphoma (C82-C85) | Overall | 5 | 1,10,13,18,22 | 1.09 | 0.86 | 1.32 | 0 (0.577) | 0.014 |
|  | < 1950 | 1 | 10 | 0.90 | 0.40 | 1.90 | - | - |
|  | 1950 – ‘70 | 1 | 22 | 1.02 | 0.68 | 1.53 | - | - |
|  | > 1970 | 3 | 1,13,18 | 1.19 | 0.79 | 1.60 | 23.5 (0.270) | 0.076 |
| Multiple myeloma (C90) | Overall | 4 | 10,13,16,23 | 1.11 | 0.85 | 1.38 | 0 (0.888) | 0 |
|  | < 1950 | 2 | 10,16 | 0.99 | 0.25 | 1.73 | 0 (0.492) | 0 |
|  | 1950 – ‘70 | 1 | 23 | 1.13 | 0.81 | 1.53 | - | - |
|  | > 1970 | 1 | 13 | 1.14 | 0.64 | 1.89 | - | - |
| Leukemia (C91-C95) | Overall | 9 | 1,6,8,10,13,16,18,20,23 | 1.05 | 0.66 | 1.45 | 35.4 (0.135) | 0.248 |
|  | < 1950 | 2 | 10,16 | 0.66 | 0.06 | 1.27 | 70.4 (0.066) | 0.049 |
|  | 1950 – ‘70 | 3 | 8,20,23 | 1.65 | 0.21 | 3.09 | 50.3 (0.134) | 1.387 |
|  | > 1970 | 4 | 1,6,13,18 | 0.92 | 0.68 | 1.15 | 0 (0.566) | 0 |
|  |  |  |  |  |  |  |  |  |
| **Cancer mortality** |  |  |  |  |  |  |  |  |
| All cancer (C00-C97) | Overall | 17 | 2,3,4,5,6,8,9,11,12,13,14,15,17,19,21,24,25 | 0.97 | 0.89 | 1.05 | 92.1 (<0.001) | 0.021 |
|  | < 1950 | 7 | 5,9,12,14,19,21,24 | 1.03 | 0.93 | 1.13 | 69.0 (0.004) | 0.010 |
|  | 1950 – ‘70 | 4 | 4,8,15,25 | **1.15** | **1.12** | **1.19** | 0 (0.562) | 0 |
|  | > 1970 | 6 | 2,3,6,11,13,17 | **0.81** | **0.70** | **0.92** | 90.4 (<0.001) | 0.012 |
| Buccal cavity and pharynx (C00-C14) | Overall | 6 | 3,5,9,11,14,17 | 0.97 | 0.68 | 1.26 | 68.4 (0.007) | 0.044 |
|  | < 1950 | 3 | 5,9,14 | 1.15 | 0.75 | 1.55 | 0 (0.484) | 0 |
|  | 1950 – ‘70 | 0 | - | - |  | - | - | - |
|  | > 1970 | 3 | 3,11,17 | 0.84 | 0.37 | 1.31 | 85.4 (0.001) | 0.095 |
| Digestive (C15-C26) | Overall | 4 | 11,17,21,25 | 0.98 | 0.71 | 1.24 | 61.1 (0.052) | 0.045 |
|  | < 1950 | 1 | 21 | **0.80** | **0.68** | **0.94** | - | - |
|  | 1950 – ‘70 | 1 | 25 | 1.38 | 0.98 | 1.89 | - | - |
|  | > 1970 | 2 | 11,17 | 0.87 | 0.69 | 1.06 | 0 (0.563) | 0 |
| Esophagus (C15) | Overall | 7 | 3,4,5,8,9,17,25 | 0.93 | 0.64 | 1.23 | 73.3 (0.001) | 0.074 |
|  | < 1950 | 2 | 5,9 | 0.67 | 0.21 | 1.13 | 0 (0.411) | 0 |
|  | 1950 – ‘70 | 3 | 4,8,25 | 1.14 | 0.49 | 1.79 | 75.0 (0.018) | 0.186 |
|  | > 1970 | 2 | 3,17 | 0.86 | 0.60 | 1.11 | 31.6 (0.227) | 0 |
| Stomach (C16) | Overall | 11 | 2,3,4,5,6,8,9,14,17,24,25 | 0.94 | 0.80 | 1.08 | 48.8 (0.034) | 0.009 |
|  | < 1950 | 4 | 5,9,14,24 | 0.98 | 0.73 | 1.24 | 0 (0.796) | 0 |
|  | 1950 – ‘70 | 3 | 4,8,25 | 0.95 | 0.55 | 1.35 | 73.9 (0.022) | 0.067 |
|  | > 1970 | 4 | 2,3,6,17 | 0.85 | 0.59 | 1.11 | 56.7 (0.074) | 0.020 |
| Colon (C18) | Overall | 8 | 3,4,5,6,9,17,24,25 | 1.07 | 0.78 | 1.35 | 67.2 (0.003) | 0.106 |
|  | < 1950 | 3 | 5,9,24 | 1.12 | 0.67 | 1.57 | 69.2 (0.039) | 0.105 |
|  | 1950 – ‘70 | 2 | 4,25 | 1.19 | 0 | 2.40 | 87.4 (0.005) | 0.672 |
|  | > 1970 | 3 | 3,6,17 | 0.95 | 0.66 | 1.24 | 50.5 (0.133) | 0.022 |
| Colorectal combined (C18-C21) | Overall | 4 | 2,7,9,14 | 1.47 | 0.52 | 2.42 | 86.5 (<0.001) | 0.869 |
|  | < 1950 | 2 | 9,14 | 1.18 | 0.47 | 1.90 | 63.0 (0.100) | 0.204 |
|  | 1950 – ‘70 | 1 | 7 | **2.79** | **1.98** | **3.81** | - | - |
|  | > 1970 | 1 | 2 | 0.65 | 0.34 | 1.14 | - | - |
| Rectum combined (C19-C21) | Overall | 9 | 3,4,5,6,8,9,17,24,25 | **1.35** | **1.12** | **1.59** | 0 (0.692) | 0.029 |
|  | < 1950 | 3 | 5,9,24 | 1.24 | 0.59 | 1.89 | 0 (0.380) | 0.201 |
|  | 1950 – ‘70 | 3 | 4,8,25 | **1.52** | **1.28** | **1.75** | 0 (0.766) | 0 |
|  | > 1970 | 3 | 3,6,17 | 1.23 | 0.86 | 1.60 | 0 (0.702) | 0 |
| Rectum (C19-C21) | Overall | 6 | 4,5,6,8,9,24 | **1.36** | **1.07** | **1.65** | 0 (0.553) | 0.038 |
|  | < 1950 | 3 | 5,9,24 | 1.24 | 0.59 | 1.89 | 0 (0.380) | 0.201 |
|  | 1950 – ‘70 | 2 | 4,8 | **1.49** | **1.25** | **1.73** | 0 (0.682) | 0 |
|  | > 1970 | 1 | 6 | 1.21 | 0.30 | 3.10 | - | - |
| Liver and biliary passages (C22-C24) | Overall | 3 | 5,9,25 | 0.95 | 0.47 | 1.43 | 0 (0.790) | 0 |
|  | < 1950 | 2 | 5,9 | 0.95 | 0.44 | 1.46 | 0 (0.495) | 0 |
|  | 1950 – ‘70 | 1 | 25 | 0.98 | 0.11 | 3.52 | - | - |
|  | > 1970 | 0 | - | - | - | - | - | - |
| Liver (C22) | Overall | 5 | 2,3,4,17,24 | 0.84 | 0.56 | 1.11 | 75.5 (0.003) | 0.035 |
|  | < 1950 | 1 | 24 | 1.49 | 0.41 | 3.81 | - | - |
|  | 1950 – ‘70 | 1 | 4 | 0.84 | 0.10 | 3.05 | - | - |
|  | > 1970 | 3 | 2,3,17 | 0.81 | 0.47 | 1.15 | 86.4 (0.001) | 0.059 |
| Pancreas (C25) | Overall | 8 | 3,4,5,9,14,17,24,25 | 0.97 | 0.73 | 1.22 | 54.9 (0.030) | 0.041 |
|  | < 1950 | 4 | 5,9,14,24 | 0.96 | 0.68 | 1.24 | 0 (0.816) | 0 |
|  | 1950 – ‘70 | 2 | 4,25 | 0.93 | 0 | 1.93 | 84.1 (0.012) | 0.376 |
|  | > 1970 | 2 | 3,17 | 0.93 | 0.25 | 1.62 | 88.3 (0.003) | 0.206 |
| Respiratory (C30-C39) | Overall | 4 | 11,12,21,25 | 0.90 | 0.73 | 1.08 | 0 (0.933) | 0 |
|  | < 1950 | 2 | 12,21 | 0.88 | 0.67 | 1.09 | 0 (0.898) | 0 |
|  | 1950 – ‘70 | 1 | 25 | 0.94 | 0.62 | 1.36 | - | - |
|  | > 1970 | 1 | 11 | 1.12 | 0.45 | 2.30 | - | - |
| Larynx (C32) | Overall | 3 | 4,5,9 | 0.59 | 0.06 | 1.12 | 0 (0.553) | 0 |
|  | < 1950 | 2 | 5,9 | 0.64 | 0.05 | 1.23 | 0 (0.449) | 0 |
|  | 1950 – ‘70 | 1 | 4 | 0.37 | 0.01 | 2.06 | - | - |
|  | > 1970 | 0 | - | - | - | - | - | - |
| Trachea and lung combined (C33-C34) | Overall | 11 | 2,3,4,5,6,8,9,14,15,17,24 | 0.98 | 0.86 | 1.11 | 72.1 (<0.001) | 0.025 |
|  | < 1950 | 4 | 5,9,14,24 | 1.07 | 0.90 | 1.24 | 15.4 (0.315) | 0.011 |
|  | 1950 – ‘70 | 3 | 4,8,15 | 1.09 | 0.87 | 1.32 | 11.9 (0.321) | 0.021 |
|  | > 1970 | 4 | 2,3,6,17 | **0.84** | **0.71** | **0.98** | 62.3 (0.047) | 0.007 |
| Trachea and lung (C33, C34) | Overall | 9 | 4,5,6,8,9,14,15,17,24 | 1.05 | 0.96 | 1.14 | 27.4 (0.201) | 0.006 |
|  | < 1950 | 4 | 5,9,14,24 | 1.07 | 0.90 | 1.24 | 15.4 (0.315) | 0.011 |
|  | 1950 – ‘70 | 3 | 4,8,15 | 1.09 | 0.87 | 1.32 | 11.9 (0.321) | 0.021 |
|  | > 1970 | 2 | 6,17 | 0.93 | 0.78 | 1.07 | 0 (0.796) | 0 |
| Skin combined (C43-C44) | Overall | 7 | 3,4,5,6,9,14,17 | 0.87 | 0.59 | 1.15 | 0 (0.927) | 0 |
|  | < 1950 | 3 | 5,9,14 | 1.02 | 0.52 | 1.51 | 0 (0.909) | 0 |
|  | 1950 – ‘70 | 1 | 4 | 0.73 | 0.09 | 2.63 | - | - |
|  | > 1970 | 3 | 3,6,17 | 0.80 | 0.45 | 1.16 | 0 (0.707) | 0 |
| Skin (C43, C44) | Overall | 5 | 3,5,9,14,17 | 0.89 | 0.59 | 1.19 | 0 (0.823) | 0 |
|  | < 1950 | 3 | 5,9,14 | 1.02 | 0.52 | 1.51 | 0 (0.909) | 0 |
|  | 1950 – ‘70 | 0 | - | - | - | - | - | - |
|  | > 1970 | 2 | 3,17 | 0.82 | 0.44 | 1.20 | 0 (0.456) | 0 |
| Breast (C50) | Overall | 3 | 3,8,17 | 3.08 | 0 | 7.15 | 10.5 (0.327) | 11.926 |
|  | < 1950 | 0 | - | - | - | - | - | - |
|  | 1950 – ‘70 | 1 | 8 | 1.43 | 0.46 | 3.34 | - | - |
|  | > 1970 | 2 | 3,17 | 4.02 | 0 | 10.5 | 59.6 (0.116) | 20.685 |
| Prostate (C61) | Overall | 9 | 3,4,5,8,9,14,17,24,25 | 1.04 | 0.86 | 1.22 | 54.8 (0.024) | 0.028 |
|  | < 1950 | 4 | 5,9,14,24 | 1.16 | 0.93 | 1.39 | 0 (0.588) | 0 |
|  | 1950 – ‘70 | 3 | 4,8,25 | 1.06 | 0.94 | 1.18 | 0 (0.422) | 0 |
|  | > 1970 | 2 | 3,17 | 0.79 | 0.26 | 1.32 | 83.2 (0.015) | 0.104 |
| Kidney combined (C64-C66) | Overall | 8 | 3,4,5,8,9,14,24,25 | 1.18 | 0.42 | 1.94 | 76.1 (<0.001) | 1.008 |
|  | < 1950 | 4 | 5,9,14,24 | 1.55 | 0 | 3.17 | 82.1 (0.001) | 2.457 |
|  | 1950 – ‘70 | 3 | 4,8,25 | 1.12 | 0.61 | 1.62 | 63.3 (0.066) | 0.086 |
|  | > 1970 | 1 | 3 | 0.63 | 0.30 | 1.16 | - | - |
| Kidney (C64-C66) | Overall | 7 | 4,5,8,9,14,24,25 | 1.28 | 0.40 | 2.15 | 75.8 (<0.001) | 1.174 |
|  | < 1950 | 4 | 5,9,14,24 | 1.55 | 0 | 3.17 | 82.1 (0.001) | 2.457 |
|  | 1950 – ‘70 | 3 | 4,8,25 | 1.12 | 0.61 | 1.62 | 63.3 (0.066) | 0.086 |
|  | > 1970 | 0 | - | - | - | - | - | - |
| Bladder combined (C67-C68) | Overall | 9 | 3,4,5,6,8,9,14,17,25 | 1.44 | 0.82 | 2.06 | 74.4 (<0.001) | 0.673 |
|  | < 1950 | 3 | 5,9,14 | 1.39 | 0 | 2.99 | 90.4 (<0.001) | 1.718 |
|  | 1950 – ‘70 | 3 | 4,8,25 | 1.61 | 0.49 | 2.72 | 50.2 (0.135) | 0.815 |
|  | > 1970 | 3 | 3,6,17 | 1.42 | 0.44 | 2.40 | 64.5 (0.060) | 0.491 |
| Bladder (C67) | Overall | 7 | 3,4,5,6,14,17,25 | **1.72** | **1.05** | **2.38** | 45.6 (0.088) | 0.528 |
|  | < 1950 | 2 | 5,14 | 2.02 | 0.19 | 3.86 | 4.0 (0.307) | 1.397 |
|  | 1950 – ‘70 | 2 | 4,25 | 2.03 | 0.48 | 3.57 | 25.6 (0.246) | 0.998 |
|  | > 1970 | 3 | 3,6,17 | 1.42 | 0.44 | 2.40 | 64.5 (0.060) | 0.491 |
| Brain combined (C70-C72) | Overall | 9 | 4,5,6,9,14,17,21,24,25 | 1.42 | 0.90 | 1.93 | 61.3 (0.008) | 0.418 |
|  | < 1950 | 5 | 5,9,14,21,24 | 1.47 | 0.74 | 2.20 | 61.9 (0.033) | 0.469 |
|  | 1950 – ‘70 | 2 | 4,25 | **2.10** | **1.47** | **2.74** | 0 (0.826) | 0 |
|  | > 1970 | 2 | 6,17 | 0.66 | 0.25 | 1.07 | 0 (0.960) | 0 |
| Brain (C70-C72) | Overall | 4 | 4,5,6,9 | 1.37 | 0.57 | 2.16 | 75.3 (0.007) | 0.501 |
|  | < 1950 | 2 | 5,9 | 1.33 | 0 | 2.76 | 88.3 (0.003) | 0.970 |
|  | 1950 – ‘70 | 1 | 4 | **2.01** | **1.10** | **3.37** | - | - |
|  | > 1970 | 1 | 6 | 0.68 | 0.10 | 2.40 | - | - |
| Brain (C71) | Overall | 5 | 14,17,21,24,25 | 1.48 | 0.71 | 2.26 | 48.2 (0.103) | 0.507 |
|  | < 1950 | 3 | 14,21,24 | 1.61 | 0.61 | 2.62 | 0 (0.46) | 0.445 |
|  | 1950 – ‘70 | 1 | 25 | 2.36 | 0.86 | 5.13 | - | - |
|  | > 1970 | 1 | 17 | 0.66 | 0.35 | 1.13 | - | - |
| Lymphohematopoietic (C81-C96) | Overall | 7 | 2,3,6,17,21,24,25 | **0.76** | **0.61** | **0.91** | 0 (0.425) | 0 |
|  | < 1950 | 2 | 21,24 | **0.60** | **0.30** | **0.90** | 0 (0.388) | 0 |
|  | 1950 – ‘70 | 1 | 25 | 0.55 | 0.18 | 1.29 | - | - |
|  | > 1970 | 4 | 2,3,6,17 | 0.83 | 0.66 | 1.01 | 0 (0.852) | 0 |
| Non-Hodgkin lymphoma combined (C82-C85) | Overall | 4 | 4,5,9,17 | 1.31 | 0.92 | 1.70 | 0 (0.446) | 0 |
|  | < 1950 | 2 | 5,9 | 1.41 | 0.96 | 1.86 | 0 (0.989) | 0 |
|  | 1950 – ‘70 | 1 | 4 | 2.04 | 0.42 | 5.96 | - | - |
|  | > 1970 | 1 | 17 | 0.65 | 0.13 | 1.90 | - | - |
| Leukemia combined (C91-C95) | Overall | 6 | 2,4,5,8,9,17 | 1.04 | 0.88 | 1.19 | 0 (0.459) | 0 |
|  | < 1950 | 2 | 5,9 | 1.02 | 0.59 | 1.44 | 23.5 (0.253) | 0.027 |
|  | 1950 – ‘70 | 2 | 4,8 | 1.11 | 0.92 | 1.30 | 0 (0.660) | 0 |
|  | > 1970 | 2 | 2,17 | 0.78 | 0.39 | 1.16 | 0 (0.558) | 0 |

*Study IDs* IDs of included studies in this meta-analysis as depicted in Table 1, *mRR* meta-relative risk assessed with inverse-variance random effects meta-analysis and Paule-Mandel heterogeneity variance estimator τ², *CI* confidence interval, *p value* p value of heterogeneity test

**Table S5** Meta-relative risk estimates (mRR) and 95% confidence intervals (CI) assessed with random effects models and Paule-Mandel heterogeneity variance estimator (τ²) stratified by region

| Disease (ICD-10 code) | Region | # Studies | Study IDs | mRR | 95% CI | | I² (p value) | τ² |
| --- | --- | --- | --- | --- | --- | --- | --- | --- |
| **Cancer incidence** |  |  |  |  |  |  |  |  |
| All cancer (C00-C97) | Overall | 9 | 1,6,8,10,13,16,18,22,23 | 1.00 | 0.93 | 1.07 | 91.27 (<0.001) | 0.010 |
|  | USA + Canada | 3 | 8,10,18 | 1.01 | 0.84 | 1.18 | 96.94 (<0.001) | 0.021 |
|  | Europe | 3 | 16,22,23 | 0.98 | 0.82 | 1.14 | 90.67 (<0.001) | 0.018 |
|  | KOR/AUS/NZL | 3 | 1,6,13 | 1.02 | 0.94 | 1.10 | 58.70 (0.089) | 0.003 |
| Buccal cavity and pharynx (C00-C14) | Overall | 4 | 10,13,18,23 | 0.87 | 0.72 | 1.02 | 41.84 (0.161) | 0.003 |
|  | USA + Canada | 2 | 10,18 | 0.79 | 0.41 | 1.16 | 52.50 (0.147) | 0.033 |
|  | Europe | 1 | 23 | 0.92 | 0.62 | 1.31 | - | - |
|  | KOR/AUS/NZL | 1 | 13 | 0.95 | 0.71 | 1.23 | - | - |
| Esophagus (C15) | Overall | 8 | 1,6,8,10,13,16,18,23 | 1.06 | 0.76 | 1.36 | 65.72 (0.005) | 0.088 |
|  | USA + Canada | 3 | 8,10,18 | 1.22 | 0.55 | 1.89 | 85.55 (0.001) | 0.256 |
|  | Europe | 2 | 16,23 | 0.98 | 0.66 | 1.30 | 0 (0.982) | 0 |
|  | KOR/AUS/NZL | 3 | 1,6,13 | 0.82 | 0.43 | 1.21 | 0 (0.587) | 0 |
| Stomach (C16) | Overall | 8 | 1,6,8,10,13,16,18,23 | 1.08 | 0.80 | 1.35 | 71.09 (0.001) | 0.109 |
|  | USA + Canada | 3 | 8,10,18 | 0.98 | 0.46 | 1.50 | 87.91 (<0.001) | 0.156 |
|  | Europe | 2 | 16,23 | 1.45 | 0.67 | 2.23 | 74.60 (0.047) | 0.281 |
|  | KOR/AUS/NZL | 3 | 1,6,13 | 0.93 | 0.77 | 1.10 | 0 (0.894) | 0 |
| Colon (C18) | Overall | 6 | 6,10,13,16,18,23 | **1.11** | **1.00** | **1.21** | 19.62 (0.285) | 0 |
|  | USA + Canada | 2 | 10,18 | 1.15 | 0.94 | 1.35 | 0 (0.832) | 0 |
|  | Europe | 2 | 16,23 | 1.07 | 0.83 | 1.31 | 38.41 (0.203) | 0.015 |
|  | KOR/AUS/NZL | 2 | 6,13 | 0.93 | 0.43 | 1.44 | 75.95 (0.041) | 0.092 |
| Colorectal combined (C18-C21) | Overall | 5 | 1,10,13,22,23 | 1.08 | 1.00 | 1.16 | 0 (0.539) | 0 |
|  | USA + Canada | 1 | 10 | 1.06 | 0.74 | 1.48 | - | - |
|  | Europe | 2 | 22,23 | 1.04 | 0.92 | 1.16 | 18.4 (0.268) | 0.002 |
|  | KOR/AUS/NZL | 2 | 1,13 | 1.15 | 0.98 | 1.32 | 0 (0.329) | 0.004 |
| Rectum combined (C19-C21) | Overall | 8 | 6,8,10,13,16,18,22,23 | 1.09 | 0.99 | 1.19 | 0 (0.819) | 0 |
|  | USA + Canada | 3 | 8,10,18 | 1.11 | 0.96 | 1.26 | 0 (0.406) | 0 |
|  | Europe | 3 | 16,22,23 | 1.05 | 0.89 | 1.20 | 0 (0.557) | 0 |
|  | KOR/AUS/NZL | 2 | 6,13 | 1.18 | 0.91 | 1.44 | 0 (0.942) | 0 |
| Rectum (C19-C21) | Overall | 5 | 6,8,10,16,23 | 1.09 | 0.97 | 1.20 | 0 (0.732) | 0 |
|  | USA + Canada | 2 | 8,10 | 1.15 | 0.99 | 1.31 | 0 (0.624) | 0 |
|  | Europe | 2 | 16,23 | 1.02 | 0.85 | 1.19 | 0 (0.403) | 0 |
|  | KOR/AUS/NZL | 1 | 6 | 1.15 | 0.50 | 2.20 | - | - |
| Liver (C22) | Overall | 4 | 1,13,18,23 | **0.81** | **0.65** | **0.98** | 16.6 (0.309) | 0 |
|  | USA + Canada | 1 | 18 | 0.74 | 0.32 | 1.46 | - | - |
|  | Europe | 1 | 23 | 0.91 | 0.59 | 1.34 | - | - |
|  | KOR/AUS/NZL | 2 | 1,13 | 0.76 | 0.50 | 1.03 | 66.9 (0.082) | 0.013 |
| Pancreas (C25) | Overall | 8 | 1,6,10,13,16,18,22,23 | 1.08 | 0.88 | 1.28 | 39.8 (0.114) | 0.021 |
|  | USA + Canada | 2 | 10,18, | 0.71 | 0.25 | 1.18 | 49.0 (0.162) | 0.029 |
|  | Europe | 3 | 16,22,23 | **1.23** | **1.01** | **1.45** | 0 (0.530) | 0.005 |
|  | KOR/AUS/NZL | 3 | 1,6,13 | 1.05 | 0.71 | 1.39 | 0 (0.908) | 0 |
| Larynx (C32) | Overall | 5 | 1,10,13,18,23 | 0.88 | 0.66 | 1.10 | 0 (0.550) | 0 |
|  | USA + Canada | 2 | 10,18 | 0.77 | 0.43 | 1.12 | 0 (0.514) | 0 |
|  | Europe | 1 | 23 | 1.06 | 0.72 | 1.50 | - | - |
|  | KOR/AUS/NZL | 2 | 1,13 | 0.78 | 0.31 | 1.24 | 0 (0.425) | 0 |
| Trachea and lung combined (C33-C34) | Overall | 8 | 1,6,8,10,13,16,18,23 | 0.91 | 0.78 | 1.03 | 87.7 (<0.001) | 0 |
|  | USA + Canada | 3 | 8,10,18 | 0.93 | 0.63 | 1.23 | 95.5 (<0.001) | 0.062 |
|  | Europe | 2 | 16,23 | 0.95 | 0.85 | 1.06 | 22.9 (0.255) | 0 |
|  | KOR/AUS/NZL | 3 | 1,6,13 | **0.83** | **0.68** | **0.98** | 0 (0.417) | 0 |
| Trachea and lung (C33, C34) | Overall | 7 | 1,6,8,10,13,16,23 | 0.97 | 0.85 | 1.08 | 71.1 (0.002) | 0.011 |
|  | USA + Canada | 2 | 8,10 | **1.14** | **1.06** | **1.22** | 0 (0.357) | 0 |
|  | Europe | 2 | 16,23 | 0.95 | 0.85 | 1.06 | 22.9 (0.255) | 0 |
|  | KOR/AUS/NZL | 3 | 1,6,13 | **0.83** | **0.68** | **0.98** | 0 (0.417) | 0 |
| Skin combined (C43-C44) | Overall | 7 | 6,10,13,16,18,22,23 | 1.16 | 0.98 | 1.35 | 74.2 (0.001) | 0.042 |
|  | USA + Canada | 2 | 10,18 | 1.17 | 0.97 | 1.38 | 0 (0.947) | 0 |
|  | Europe | 3 | 16,22,23 | 1.02 | 0.67 | 1.36 | 85.6 (0.001) | 0.079 |
|  | KOR/AUS/NZL | 2 | 6,13 | **1.43** | **1.27** | **1.58** | 0 (0.571) | 0 |
| Malignant melanoma of skin (C43) | Overall | 6 | 6,10,13,16,22,23 | 1.19 | 0.89 | 1.48 | 78.8 (<0.001) | 0.090 |
|  | USA + Canada | 1 | 10 | 1.20 | 0.60 | 2.30 | - | - |
|  | Europe | 3 | 16,22,23 | 1.00 | 0.40 | 1.60 | 89.6 (<0.001) | 0.237 |
|  | KOR/AUS/NZL | 2 | 6,13 | **1.43** | **1.27** | **1.58** | 0 (0.571) | 0 |
| Other malignant neoplasms of the skin (C44) | Overall | 4 | 16,18,22,23 | 1.10 | 0.90 | 1.30 | 63.2 (0.043) | 0.026 |
|  | USA + Canada | 1 | 18 | 1.17 | 0.95 | 1.42 | - | - |
|  | Europe | 3 | 16,23,23 | 1.07 | 0.79 | 1.35 | 72.9 (0.025) | 0.043 |
|  | KOR/AUS/NZL | 0 | - | - | - | - | - | - |
| Breast (C50) | Overall | 4 | 8,10,13,18 | 1.23 | 0.27 | 2.19 | 28.6 (0.240) | 0.513 |
|  | USA + Canada | 3 | 8,10,18 | 0.75 | 0.18 | 1.32 | 0 (0.561) | 0 |
|  | Europe | 0 | - | - | - | - | - | - |
|  | KOR/AUS/NZL | 1 | 13 | 2.49 | 0.81 | 5.82 | - | - |
| Prostate (C61) | Overall | 9 | 1,6,8,10,13,16,18,22,23 | 1.10 | 0.97 | 1.22 | 75.0 (<0.001) | 0.025 |
|  | USA + Canada | 3 | 8,10,18 | 1.14 | 0.93 | 1.35 | 62.2 (0.071) | 0.027 |
|  | Europe | 3 | 16,22,23 | 0.99 | 0.70 | 1.27 | 90.1 (<0.001) | 0.057 |
|  | KOR/AUS/NZL | 3 | 1,6,13 | **1.23** | **1.11** | **1.34** | 0 (0.903) | 0 |
| Testis (C62) | Overall | 5 | 6,13,18,22,23 | 1.26 | 0.87 | 1.65 | 77.0 (0.002) | 0.141 |
|  | USA + Canada | 1 | 18 | **1.60** | **1.20** | **2.09** | - | - |
|  | Europe | 2 | 22,23 | 0.87 | 0.17 | 1.58 | 86.4 (0.007) | 0.204 |
|  | KOR/AUS/NZL | 2 | 6,13 | **1.47** | **1.10** | **1.83** | 0 (0.865) | 0 |
| Urinary tract (C64-C68) | Overall | 3 | 8,10,13 | 1.07 | 0.92 | 1.22 | 41.4 (0.182) | 0.007 |
|  | USA + Canada | 2 | 8,10 | **1.15** | **1.05** | **1.24** | 0 (0.533) | 0 |
|  | Europe | 0 | - | - | - | - | - | - |
|  | KOR/AUS/NZL | 1 | 13 | 0.91 | 0.69 | 1.17 | - | - |
| Kidney combined (C64-C66) | Overall | 8 | 1,6,8,10,13,16,18,23 | 0.98 | 0.75 | 1.20 | 62.6 (0.009) | 0.053 |
|  | USA + Canada | 3 | 8,10,18 | 0.95 | 0.54 | 1.37 | 79.8 (0.007) | 0.088 |
|  | Europe | 2 | 16,23 | 0.87 | 0.58 | 1.16 | 58.0 (0.123) | 0.016 |
|  | KOR/AUS/NZL | 3 | 1,6,13 | 1.11 | 0.61 | 1.61 | 44.2 (0.166) | 0.105 |
| Kidney (C64-C66) | Overall | 4 | 6,8,10,16 | 0.89 | 0.49 | 1.29 | 74.8 (0.008) | 0.080 |
|  | USA + Canada | 2 | 8,16 | 0.99 | 0.28 | 1.70 | 78.3 (0.032) | 0.195 |
|  | Europe | 1 | 16 | 0.57 | 0.21 | 1.23 | - | - |
|  | KOR/AUS/NZL | 1 | 6 | 0.57 | 0.10 | 2.10 | - | - |
| Bladder combined (C67-C68) | Overall | 7 | 1,6,8,10,13,18,22 | **1.14** | **1.04** | **1.23** | 0 (0.592) | 0 |
|  | USA + Canada | 3 | 8,10,18 | **1.14** | **1.04** | **1.25** | 0 (0.577) | 0 |
|  | Europe | 1 | 22 | 1.14 | 0.89 | 1.48 | - | - |
|  | KOR/AUS/NZL | 3 | 1,6,13 | 1.15 | 0.69 | 1.61 | 36.9 (0.205) | 0.075 |
| Bladder (C67) | Overall | 6 | 1,6,10,13,18,22 | **1.18** | **1.01** | **1.34** | 0 (0.492) | 0.005 |
|  | USA + Canada | 2 | 10,18 | **1.27** | **1.04** | **1.50** | 0 (0.800) | 0 |
|  | Europe | 1 | 22 | 1.14 | 0.89 | 1.48 | - | - |
|  | KOR/AUS/NZL | 3 | 1,6,13 | 1.15 | 0.69 | 1.61 | 36.9 (0.205) | 0.075 |
| Brain combined (C70-C72) | Overall | 7 | 1,6,10,13,16,18,23 | **0.81** | **0.65** | **0.98** | 6.2 (0.380) | 0 |
|  | USA + Canada | 2 | 10,18 | 0.65 | 0.28 | 1.02 | 23.5 (0.253) | 0 |
|  | Europe | 2 | 16,23 | 0.89 | 0.67 | 1.10 | 0 (0.452) | 0 |
|  | KOR/AUS/NZL | 3 | 1,6,13 | 0.78 | 0.44 | 1.12 | 2.6 (0.658) | 0 |
| Brain (C70-C72) | Overall | 5 | 1,6,10,13,16 | 0.87 | 0.57 | 1.16 | 0 (0.489) | 0 |
|  | USA + Canada | 1 | 10 | 1.10 | 0.30 | 2.90 | - | - |
|  | Europe | 1 | 16 | 1.16 | 0.50 | 2.28 | - | - |
|  | KOR/AUS/NZL | 3 | 1,6,13 | 0.78 | 0.44 | 1.12 | 2.6 (0.358) | 0 |
| Thyroid (C73) | Overall | 5 | 1,10,13,18,23 | 1.26 | 0.98 | 1.54 | 0 (0.623) | 0.011 |
|  | USA + Canada | 2 | 10,18 | 1.64 | 0.98 | 2.29 | 0 (0.389) | 0.042 |
|  | Europe | 1 | 23 | 1.28 | 0.75 | 2.05 | - | - |
|  | KOR/AUS/NZL | 2 | 1,13 | 1.07 | 0.71 | 1.42 | 0 (0.662) | 0 |
| Lymphohematopoietic (C81-C96) | Overall | 4 | 1,13,16,18 | 0.90 | 0.63 | 1.17 | 76.1 (0.006) | 0.055 |
|  | USA + Canada | 1 | 18 | **0.68** | **0.54** | **0.85** | - | - |
|  | Europe | 1 | 16 | 0.73 | 0.43 | 1.16 | - | - |
|  | KOR/AUS/NZL | 2 | 1,13 | 1.10 | 0.73 | 1.46 | 56.0 (0.132) | 0.047 |
| Hodgkin's disease (C81) | Overall | 4 | 10,13,16,18 | 0.84 | 0.44 | 1.24 | 0 (0.906) | 0 |
|  | USA + Canada | 2 | 10,18 | 0.76 | 0.27 | 1.26 | 0 (0.914) | 0 |
|  | Europe | 1 | 16 | 1.39 | 0.17 | 5.00 | - | - |
|  | KOR/AUS/NZL | 1 | 13 | 0.91 | 0.34 | 1.99 | - | - |
| Non-Hodgkin lymphoma combined (C82-C85) | Overall | 6 | 1,10,13,16,18,22 | 1.05 | 0.83 | 1.28 | 0 (0.484) | 0.018 |
|  | USA + Canada | 2 | 10,18 | 1.02 | 0.60 | 1.44 | 0 (0.669) | 0 |
|  | Europe | 2 | 16,22 | 0.92 | 0.57 | 1.27 | 4.7 (0.306) | 0 |
|  | KOR/AUS/NZL | 2 | 1,13 | 1.28 | 0.60 | 1.97 | 61.8 (0.105) | 0.195 |
| Non-Hodgkin lymphoma (C82-C85) | Overall | 5 | 1,10,13,18,22 | 1.09 | 0.86 | 1.32 | 0 (0.577) | 0.014 |
|  | USA + Canada | 2 | 10,18 | 1.02 | 0.60 | 1.44 | 0 (0.669) | 0 |
|  | Europe | 1 | 22 | 1.02 | 0.68 | 1.53 | - | - |
|  | KOR/AUS/NZL | 2 | 1,13 | 1.28 | 0.60 | 1.97 | 61.95 (0.105) | 0.195 |
| Multiple myeloma (C90) | Overall | 4 | 10,13,16,23 | 1.11 | 0.85 | 1.38 | 0 (0.888) | 0 |
|  | USA + Canada | 1 | 10 | 0.70 | 0.10 | 2.60 | - | - |
|  | Europe | 2 | 16,23 | 1.14 | 0.83 | 1.44 | 0 (0.933) | 0 |
|  | KOR/AUS/NZL | 1 | 13 | 1.14 | 0.64 | 1.89 | - | - |
| Leukemia (C91-C95) | Overall | 9 | 1,6,8,10,13,16,18,20,23 | 1.05 | 0.66 | 1.45 | 35.4 (0.135) | 0.248 |
|  | USA + Canada | 4 | 8,10,18,20 | 1.30 | 0.22 | 2.39 | 0 (0.419) | 1.037 |
|  | Europe | 2 | 16,23 | 0.86 | 0 | 1.73 | 87.9 (0.004) | 0.303 |
|  | KOR/AUS/NZL | 3 | 1,6,13 | 1.00 | 0.71 | 1.29 | 0 (0.602) | 0 |
|  |  |  |  |  |  |  |  |  |
| **Cancer mortality** |  |  |  |  |  |  |  |  |
| All cancer (C00-C97) | Overall | 17 | 2,3,4,5,6,8,9,11,12,13,14,15,17,19,21,24,25 | 0.97 | 0.89 | 1.05 | 92.1 (<0.001) | 0.021 |
|  | USA + Canada | 9 | 4,5,8,9,14,17,19,21,25 | 1.03 | 0.94 | 1.12 | 88.7 (<0.001) | 0.014 |
|  | Europe | 4 | 3,11,15,24 | 0.96 | 0.90 | 1.02 | 0 (0.741) | 0 |
|  | KOR/AUS/NZL | 4 | 2,6,12,13 | **0.78** | **0.59** | **0.97** | 87.9 (<0.001) | 0.025 |
| Buccal cavity and pharynx (C00-C14) | Overall | 6 | 3,5,9,11,14,17 | 0.97 | 0.68 | 1.26 | 68.4 (0.007) | 0.044 |
|  | USA + Canada | 4 | 5,9,14,17 | 0.88 | 0.43 | 1.32 | 74.1 (0.009) | 0.088 |
|  | Europe | 2 | 3,11 | 1.14 | 0.89 | 1.38 | 0 (0.589) | 0 |
|  | KOR/AUS/NZL | 0 | - | - | - | - | - | - |
| Digestive (C15-C26) | Overall | 4 | 11,17,21,25 | 0.98 | 0.71 | 1.24 | 61.1 (0.052) | 0.045 |
|  | USA + Canada | 3 | 17,21,25 | 0.98 | 0.64 | 1.32 | 72.8 (0.025) | 0.076 |
|  | Europe | 1 | 11 | 1.14 | 0.37 | 2.66 | - | - |
|  | KOR/AUS/NZL | 0 | - | - | - | - | - | - |
| Esophagus (C15) | Overall | 7 | 3,4,5,8,9,17,25 | 0.93 | 0.64 | 1.23 | 73.3 (0.001) | 0.074 |
|  | USA + Canada | 6 | 4,5,8,9,17,25 | 0.92 | 0.56 | 1.28 | 77.0 (0.001) | 0.098 |
|  | Europe | 1 | 3 | 0.93 | 0.67 | 1.27 | - | - |
|  | KOR/AUS/NZL | 0 | - | - | - | - | - | - |
| Stomach (C16) | Overall | 11 | 2,3,4,5,6,8,9,14,17,24,25 | 0.94 | 0.80 | 1.08 | 48.8 (0.034) | 0.009 |
|  | USA + Canada | 7 | 4,5,8,9,14,17,25 | 1.00 | 0.86 | 1.15 | 30.8 (0.193) | 0 |
|  | Europe | 2 | 3,24 | 1.17 | 0.84 | 1.50 | 0 (0.888) | 0 |
|  | KOR/AUS/NZL | 2 | 2,6 | **0.65** | **0.39** | **0.91** | 0 (0.335) | 0 |
| Colon (C18) | Overall | 8 | 3,4,5,6,9,17,24,25 | 1.07 | 0.78 | 1.35 | 67.2 (0.003) | 0.106 |
|  | USA + Canada | 5 | 4,5,9,17,25 | 1.16 | 0.75 | 1.58 | 74.2 (0.004) | 0.174 |
|  | Europe | 2 | 3,24 | 0.75 | 0.46 | 1.03 | 0 (0.707) | 0 |
|  | KOR/AUS/NZL | 1 | 6 | 1.19 | 0.40 | 2.60 | - | - |
| Colorectal combined (C18-C21) | Overall | 4 | 2,7,9,14 | 1.47 | 0.52 | 2.42 | 86.5 (<0.001) | 0.869 |
|  | USA + Canada | 3 | 7,9,14 | 1.75 | 0.64 | 2.85 | 87.0 (0) | 0.881 |
|  | Europe | 0 | - | - | - | - | - | - |
|  | KOR/AUS/NZL | 1 | 2 | 0.65 | 0.34 | 1.14 | - | - |
| Rectum combined (C19-C21) | Overall | 9 | 3-6,8,9,17,24,25 | **1.35** | **1.12** | **1.59** | 0 (0.692) | 0.029 |
|  | USA + Canada | 6 | 4,5,8,9,17,25 | 1.31 | 0.99 | 1.62 | 0 (0.441) | 0.061 |
|  | Europe | 2 | 3,24 | 1.58 | 0.94 | 2.22 | 0 (0.454) | 0.094 |
|  | KOR/AUS/NZL | 1 | 6 | 1.21 | 0.30 | 3.10 | - | - |
| Rectum (C19-C21) | Overall | 6 | 4,5,6,8,9,24 | **1.36** | **1.07** | **1.65** | 0 (0.553) | 0.038 |
|  | USA + Canada | 4 | 4,5,8,9 | 1.30 | 0.98 | 1.62 | 6.3 (0.362) | 0.043 |
|  | Europe | 1 | 24 | 2.07 | 0.89 | 4.08 | - | - |
|  | KOR/AUS/NZL | 1 | 6 | 1.21 | 0.30 | 3.10 | - | - |
| Liver (C22) | Overall | 5 | 2,3,4,17,24 | 0.84 | 0.56 | 1.11 | 75.5 (0.003) | 0.035 |
|  | USA + Canada | 2 | 4,17 | 0.85 | 0.33 | 1.37 | 0 (0.987) | 0 |
|  | Europe | 2 | 3,24 | 1.12 | 0.83 | 1.42 | 0 (0.629) | 0 |
|  | KOR/AUS/NZL | 1 | 2 | **0.55** | **0.41** | **0.73** | - | - |
| Pancreas (C25) | Overall | 8 | 3,4,5,9,14,17,24,25 | 0.97 | 0.73 | 1.22 | 54.9 (0.030) | 0.041 |
|  | USA + Canada | 6 | 4,5,9,14,17,25 | 0.90 | 0.60 | 1.21 | 55.0 (0.049) | 0.054 |
|  | Europe | 2 | 3,24 | 1.20 | 0.89 | 1.52 | 0 (0.350) | 0 |
|  | KOR/AUS/NZL | 0 | - | - | - | - | - | - |
| Respiratory (C30-C39) | Overall | 4 | 11,12,21,25 | 0.90 | 0.73 | 1.08 | 0 (0.933) | 0 |
|  | USA + Canada | 2 | 21,25 | 0.90 | 0.71 | 1.08 | 0 (0.759) | 0 |
|  | Europe | 1 | 11 | 1.12 | 0.45 | 2.30 | - | - |
|  | KOR/AUS/NZL | 1 | 12 | 0.84 | 0.33 | 1.71 | - | - |
| Lung combined (C33-C34) | Overall | 11 | 2,3,4,5,6,8,9,14,15,17,24 | 0.98 | 0.86 | 1.11 | 72.1 (<0.001) | 0.025 |
|  | USA + Canada | 6 | 4,5,8,9,14,17 | 1.05 | 0.95 | 1.15 | 43.3 (0.117) | 0.007 |
|  | Europe | 3 | 3,15,24 | 0.99 | 0.61 | 1.36 | 10.3 (0.328) | 0.063 |
|  | KOR/AUS/NZL | 2 | 2,6 | **0.64** | **0.38** | **0.90** | 30.9 (0.229) | 0 |
| Trachea and lung (C33, C34) | Overall | 9 | 4,5,6,8,9,14,15,17,24 | 1.05 | 0.96 | 1.14 | 27.4 (0.201) | 0.006 |
|  | USA + Canada | 6 | 4,5,8,9,14,17 | 1.05 | 0.95 | 1.15 | 43.3 (0.117) | 0.007 |
|  | Europe | 2 | 15,24 | 1.17 | 0.48 | 1.87 | 35.5 (0.213) | 0.151 |
|  | KOR/AUS/NZL | 1 | 6 | 0.86 | 0.40 | 1.60 | - | - |
| Skin combined (C43-C44) | Overall | 7 | 3,4,5,6,9,14,17 | 0.87 | 0.59 | 1.15 | 0 (0.927) | 0 |
|  | USA + Canada | 5 | 4,5,9,14,17 | 0.93 | 0.61 | 1.25 | 0 (0.952) | 0 |
|  | Europe | 1 | 3 | 0.65 | 0.21 | 1.51 | - | - |
|  | KOR/AUS/NZL | 1 | 6 | 0.65 | 0.10 | 2.40 | - | - |
| Skin (C43-C44) | Overall | 5 | 3,5,9,14,17 | 0.89 | 0.59 | 1.19 | 0 (0.823) | 0 |
|  | USA + Canada | 4 | 5,9,14,17 | 0.95 | 0.62 | 1.28 | 0 (0.921) | 0 |
|  | Europe | 1 | 3 | 0.65 | 0.21 | 1.51 | - | - |
|  | KOR/AUS/NZL | 0 | - | - | - | - | - | - |
| Breast (C50) | Overall | 3 | 3,8,17 | 3.08 | 0 | 7.15 | 10.5 (0.327) | 11.926 |
|  | USA + Canada | 2 | 8,17 | 4.29 | 0 | 10.14 | 31.9 (0.226) | 16.811 |
|  | Europe | 1 | 3 | 0.76 | 0.02 | 4.23 | - | - |
|  | KOR/AUS/NZL | 0 | - | - | - | - | - | - |
| Genitourinary system (C60-C68) | Overall | 3 | 8,11,21 | 1.29 | 0.23 | 2.35 | 9.6 (0.331) | 0.668 |
|  | USA + Canada | 2 | 8,21 | 1.04 | 0.91 | 1.17 | 29.5 (0.234) | 0.003 |
|  | Europe | 1 | 11 | 3.29 | 0.40 | 11.88 | - | - |
|  | KOR/AUS/NZL | 0 | - | - | - | - | - | - |
| Prostate (C61) | Overall | 9 | 3,4,5,8,9,14,17,24,25 | 1.04 | 0.86 | 1.22 | 54.8 (0.024) | 0.028 |
|  | USA + Canada | 7 | 4,5,8,9,14,17,25 | 1.08 | 0.97 | 1.18 | 0 (0.703) | 0 |
|  | Europe | 2 | 3,24 | 0.83 | 0.18 | 1.49 | 82.6 (0.016) | 0.165 |
|  | KOR/AUS/NZL | 0 | - | - | - | - | - | - |
| Kidney combined (C64-C66) | Overall | 8 | 3,4,5,8,9,14,24,25 | 1.18 | 0.42 | 1.94 | 76.1 (<0.001) | 1.008 |
|  | USA + Canada | 6 | 4,5,8,9,14,25 | 1.32 | 0.28 | 2.37 | 79.8 (<0.001) | 1.494 |
|  | Europe | 2 | 3,24 | 0.72 | 0.28 | 1.16 | 0 (0.333) | 0 |
|  | KOR/AUS/NZL | 0 | - | - |  |  | - | - |
| Kidney (C64-C66) | Overall | 7 | 4,5,8,9,14,24,25 | 1.28 | 0.40 | 2.15 | 75.8 (<0.001) | 1.174 |
|  | USA + Canada | 6 | 4,5,8,9,14,25 | 1.32 | 0.28 | 2.37 | 79.9 (<0.001) | 1.494 |
|  | Europe | 1 | 24 | 1.10 | 0.30 | 2.81 | - | - |
|  | KOR/AUS/NZL | 0 | - | - | - | - | - | - |
| Bladder combined (C67-C68) | Overall | 9 | 3,4,5,6,8,9,14,17,25 | 1.44 | 0.82 | 2.06 | 74.4 (<0.001) | 0.673 |
|  | USA + Canada | 7 | 4,5,8,9,14,17,25 | 1.50 | 0.78 | 2.21 | 79.2 (<0.001) | 0.752 |
|  | Europe | 1 | 3 | 0.73 | 0.41 | 1.21 | - | - |
|  | KOR/AUS/NZL | 1 | 6 | 2.73 | 0.30 | 9.80 | - | - |
| Bladder (C67) | Overall | 7 | 3,4,5,6,14,17,25 | **1.72** | **1.05** | **2.38** | 45.6 (0.088) | 0.528 |
|  | USA + Canada | 5 | 4,5,14,17,25 | **1.88** | **1.16** | **2.59** | 0 (0.574) | 0.437 |
|  | Europe | 1 | 3 | 0.73 | 0.41 | 1.21 | - | - |
|  | KOR/AUS/NZL | 1 | 6 | 2.73 | 0.30 | 9.80 | - | - |
| Brain combined (C70-C72) | Overall | 9 | 4-6,9,14,17,21,24,25 | 1.42 | 0.90 | 1.93 | 61.3 (0.008) | 0.418 |
|  | USA + Canada | 7 | 4,5,9,14,17,21,25 | 1.37 | 0.84 | 1.90 | 66.8 (0.006) | 0.356 |
|  | Europe | 1 | 24 | 2.79 | 0.91 | 6.54 | - | - |
|  | KOR/AUS/NZL | 1 | 6 | 0.68 | 0.10 | 2.41 | - | - |
| Brain (C70-C72) | Overall | 4 | 4,5,6,9 | 1.37 | 0.57 | 2.16 | 75.3 (0.007) | 0.501 |
|  | USA + Canada | 3 | 4,5,9 | 1.54 | 0.59 | 2.49 | 82.2 (0.004) | 0.589 |
|  | Europe | 0 | - | - | - | - | - | - |
|  | KOR/AUS/NZL | 1 | 6 | 0.68 | 0.10 | 2.40 | - | - |
| Brain (C71) | Overall | 5 | 14,17,21,24,25 | 1.48 | 0.71 | 2.26 | 48.2 (0.103) | 0.507 |
|  | USA + Canada | 4 | 14,17,21,25 | 1.21 | 0.52 | 1.89 | 40.8 (0.167) | 0.289 |
|  | Europe | 1 | 24 | 2.79 | 0.91 | 6.51 | - | - |
|  | KOR/AUS/NZL | 0 | - | - | - | - | - | - |
| Lymphohematopoietic (C81-C96) | Overall | 7 | 2,3,6,17,21,24,25 | **0.76** | **0.61** | **0.91** | 0 (0.425) | 0 |
|  | USA + Canada | 3 | 17,21,25 | **0.70** | **0.50** | **0.90** | 0 (0.494) | 0 |
|  | Europe | 2 | 3,24 | 0.80 | 0.45 | 1.15 | 66.9 (0.082) | 0.018 |
|  | KOR/AUS/NZL | 2 | 2,6 | 0.86 | 0.44 | 1.28 | 0 (0.632) | 0 |
| Leukemia combined (C91-C95) | Overall | 6 | 2,4,5,8,9,17 | 1.04 | 0.88 | 1.19 | 0 (0.459) | 0 |
|  | USA + Canada | 5 | 4,5,8,9,17 | 1.06 | 0.90 | 1.22 | 0 (0.596) | 0 |
|  | Europe | 0 | - | - | - | - | - | - |
|  | KOR/AUS/NZL | 1 | 2 | 0.66 | 0.24 | 1.44 | - | - |

*Study IDs* IDs of included studies in this meta-analysis as depicted in Table 1, *mRR* meta-relative risk assessed with inverse-variance random effects meta-analysis and Paule-Mandel heterogeneity variance estimator τ², *CI*: confidence interval, *p value* p value of heterogeneity test

**Fig. S1 Statistically non-significant meta-relative SIRs and 95% confidence intervals of cancer types stratified by start of employment**

**Fig. S2 Statistically non-significant meta-relative SMRs and 95% confidence intervals of cancer types stratified by start of employment**

**Fig. S3 Funnel plots including all studies reporting overall cancer incidence and mortality among firefighters.** The vertical line represents the fixed effect estimates of the standardized incidence ratios (SIRs) and standardized mortality ratios (SMRs). The outer dashed lines indicate the triangular region within which 95% of studies are expected to lie in the absence of biases and heterogeneity.
